# Population genomics identifies genetic signatures of carrot domestication and improvement and uncovers the origin of high-carotenoid orange carrots

**DOI:** 10.1038/s41477-023-01526-6

**Published:** 2023-09-28

**Authors:** Kevin Coe, Hamed Bostan, William Rolling, Sarah Turner-Hissong, Alicja Macko-Podgórni, Douglas Senalik, Su Liu, Romit Seth, Julien Curaba, Molla Fentie Mengist, Dariusz Grzebelus, Allen Van Deynze, Julie Dawson, Shelby Ellison, Philipp Simon, Massimo Iorizzo

**Affiliations:** 1 Plants for Human Health Institute, North Carolina State University, Kannapolis, NC USA; 2 Department of Plant and Agroecosystem Sciences, University of Wisconsin–Madison, Madison, WI USA; 3 Agricultural Research Service, Vegetable Crops Research Unit, US Department of Agriculture, Madison, WI USA; 4 Bayer Crop Science, Chesterfield, MO USA; 5 Department of Plant Biology and Biotechnology, Faculty of Biotechnology and Horticulture, University of Agriculture in Krakow, Krakow, Poland; 6 Seed Biotechnology Center, University of California, Davis, CA USA; 7 Department of Horticultural Science, North Carolina State University, Raleigh, NC USA

**Keywords:** Genetics, Plant genetics, Population genetics, Genomics

## Abstract

Here an improved carrot reference genome and resequencing of 630 carrot accessions were used to investigate carrot domestication and improvement. The study demonstrated that carrot was domesticated during the Early Middle Ages in the region spanning western Asia to central Asia, and orange carrot was selected during the Renaissance period, probably in western Europe. A progressive reduction of genetic diversity accompanied this process. Genes controlling circadian clock/flowering and carotenoid accumulation were under selection during domestication and improvement. Three recessive genes, at the *REC*, *Or* and *Y2* quantitative trait loci, were essential to select for the high α- and β-carotene orange phenotype. All three genes control high α- and β-carotene accumulation through molecular mechanisms that regulate the interactions between the carotenoid biosynthetic pathway, the photosynthetic system and chloroplast biogenesis. Overall, this study elucidated carrot domestication and breeding history and carotenoid genetics at a molecular level.

## Abstract (web summary)

This study demonstrates that orange carrot was selected during the Renaissance period, probably in western Europe, through the selection of three recessive genes that increased the provitamin A carotenoid (α- and β-carotene) content.

## Main

Carrot (*Daucus carota* L., 2*n* = 2*x* = 18) is known for being among the richest sources of dietary provitamin A carotenoids, α- and β-carotene. Carrot is grown globally, and production has risen steadily during the past 50 years^1^, with extensive adaptation to Asia, Europe and the Americas, including subtropical climates. The adaptability, nutritional value and diversification of carrot for fresh and processed markets (for example, as a natural colourant) have been the driving forces for this growth^1,2^. These attributes raise expectations that new cultivars can be developed to meet market demands and sustain expanded production under increasingly challenging environmental growing conditions. Advancing research that can enable the implementation of molecular-assisted breeding strategies is critical to support these efforts.

Carrot germplasm collections include an array of cultivars, landraces and wild carrots, which harbour a wide range of phenotypic diversity useful for breeding^3^. This crop is propagated via seed, and, as a primarily outcrossing species, hybridization within and between carrot populations is common, which facilitates gene flow within carrot germplasm^4^. It is currently well accepted that cultivated carrot germplasm can be separated into two major groups: Eastern and Western^4^. The Eastern group includes the first domesticated carrots, which were purple or yellow and originated in the region spanning Asia Minor and central Asia. According to historical records, Eastern carrots were used as a food crop in the Iranian Plateau and Persia in the tenth century^4^. The Western group, primarily represented by orange carrots, first appeared in Europe during the seventeenth century and quickly became the predominant carrot type grown and consumed globally^5^. Recent molecular studies clearly separated Wild, Eastern and Western carrot populations and indicated Eastern carrots as the progenitor of Western carrots^4,6^. Despite recent advances in understanding the genetic structure of the carrot germplasm and phylogenetic relationships between Eastern, Western and Wild carrot populations, the demographic events that characterized carrot domestication and improvement have not been investigated. Furthermore, previous studies have indicated that after carrot domestication, a genetic bottleneck was either absent or marginal^6–8^. Due to the lack of whole-genome-wide analysis, the impact of domestication and improvement on genetic diversity within carrot germplasm remains unresolved.

The selection of orange carrots in the 1500s resulted in carrots that accumulate high levels of α- and β-carotene, which, as later discovered in the 1800s and 1900s, improved the nutritional value of the crop. Indeed, ‘carotene’, the first carotenoid discovered, was initially isolated from carrot juice extracts in the 1800s and was observed to be medically active^9^. The most health benefit of carrot was demonstrated with the discovery of vitamin A in 1913^10^ and the observation that dietary carotenoids from plants can prevent vitamin A deficiency^11^. Numerous studies have demonstrated additional health benefits associated with carotenoids^12^, which probably contributed to the increased popularity of orange carrots and their consumption. For instance, carrot represents the most abundant plant source of the provitamin A carotenoids, α- and β-carotene, in the US diet today^13^. Given the importance of these compounds, increasing the α- and β-carotene content in orange carrots and studying the genetic mechanism controlling their accumulation have been primary targets of carrot breeding and genetic studies^14^. To date, two loci named *Or* and *Y2* have been associated with high α- and β-carotene and thus the appearance of an orange phenotype^7,14–17^. An *Orange like* gene homologue (*Or-like*) was identified as candidate gene controlling the *Or* locus, while several candidate genes have been identified in the genomic region associated with the *Y2* locus^7,16^. Findings from these previous studies indicate that none of the proposed candidate genes encode the biosynthetic enzymes in the carotenoid pathway. Instead, they suggest that the accumulation of high α- and β-carotene in carrots is regulated through the light-response feedback mechanism and chloroplast biogenesis^7^. The rapid increase in the popularity of orange carrot probably led to the fixation of many alleles responsible for carotenoid presence, but the roles of loci controlling carotenoid accumulation and other important domestication and improvement traits in carrot have been only partially evaluated using reduced sequence representation methods (for example, GBS and DarT)^7,18^ and biparental populations. As a result, within the *Or* and *Y2* loci, candidate genes and causal mutations have not been fully confirmed.

To advance knowledge about carrot domestication and modern breeding, we present an improved carrot genome assembly of the double haploid orange Nantes-type carrot DH1, alongside a large-scale resequencing study that represents a global collection of carrot germplasm. These data enabled us to uncover the demographic events that characterized carrot domestication and improvement and the genes that were selected during these processes. The outcomes of this study and the DH1 v.3 genome will provide improved genomic insights into traits important for carrot domestication and improvement.

## Results

### An improved carrot genome assembly and annotation

**Fig. 1:**
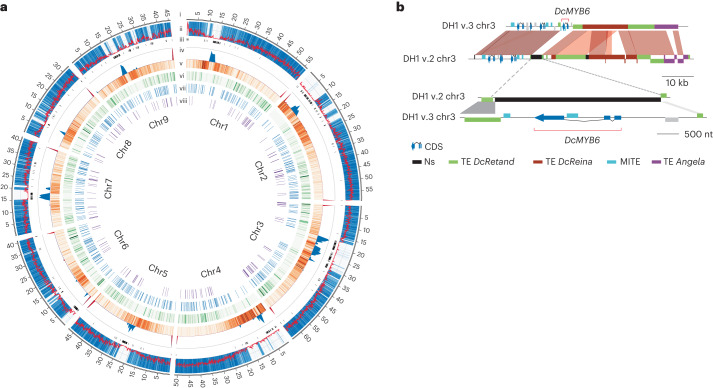
DH1 v.3 genome features and statistics. **a**, Circos display of the DH1 v.3 genomic features: (i) the chromosome (chr) coordinates in Mb; (ii) gene frequency (bin size, 100 kb) (red line) and alignments of v.2 contigs versus v.3 chromosomes (blue heat map); (iii) gaps (Ns) in the v.3 genome assembly; (iv) the telomeric repeat frequency histogram (×100) (red) and the centromeric repeat frequency histogram (blue); (v) heat map representing the distribution of *gypsy+copia* transposable elements (TEs) (bin size, 250 kb); (vi) heat map representing new v.3 genes (bin size, 250 kb); (vii) the distribution of new transcription factors; and (viii) the distribution of new resistance genes. **b**, Schematic representation of the genomic region spanning *DcMYB6* in the DH1 v.2 and v.3 genome assemblies. *DcMYB6* was not assembled at the chromosome level in DH1 v.2, probably due to complex repetitive sequences flanking the gene. The region including *DcMYB6* was fully assembled in the DH1 v.3 assembly.

**Extended Data Fig. 1:**
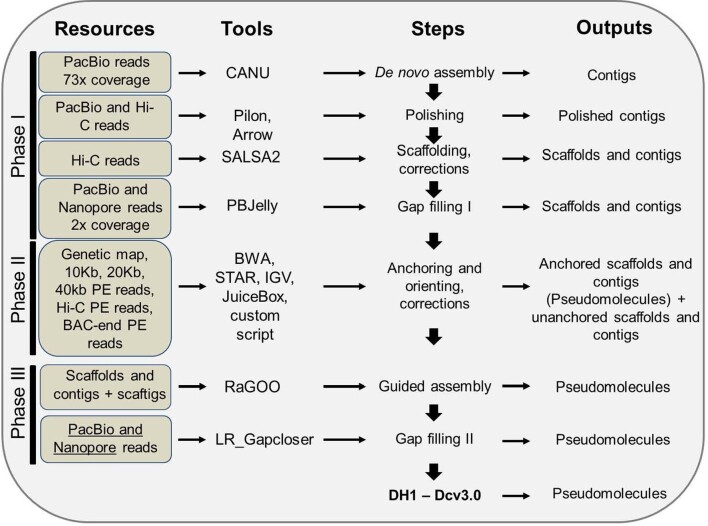
Scheme of the carrot genome assembly. In Phase I newly sequenced long reads (PacBio and Nanopore) and Hi-C reads from DH1 were used for de-novo assembly, nucleotide error correction (polishing), scaffolding and correcting chimeric sequences. These steps generated contigs and scaffolds. In Phase II, unambiguously aligned sequences from mapped molecular markers, BAC end sequences, Hi-C sequences and 10, 20 and 40 kb Illumina MPE were used to correct chimeric regions and anchor the genome assembly. These steps generated anchored and un-anchored contigs and scaffolds. In Phase III, the assembly obtained from Phase II at scaffold and contig or scaftig level was used to perform a guided genome assembly and to fill additional gaps. These steps produced the carrot DH1 assembly v3.0, that includes nine pseudomolecules or chromosomes.

**Extended Data Fig. 2:**
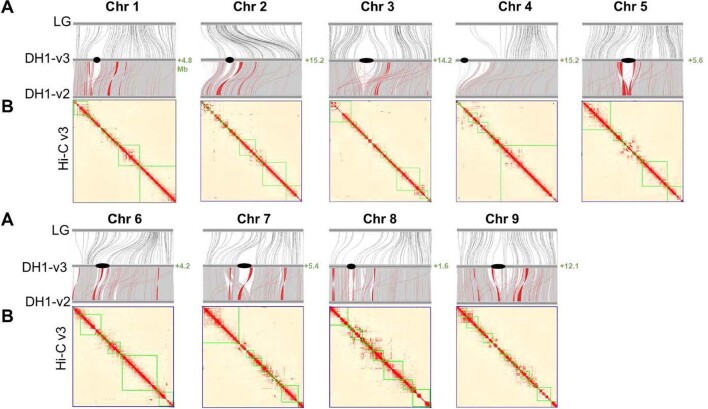
Comparison of genome assemblies with genetic map, and Hi-C data. **a**) Alignment of the integrated linkage map (LG), and the DH1-v2 assembled chromosomes with the DH1-v3 assembled chromosomes. Black dots in the DH1-v3 chromosome scheme represent the approximate location of the centromere repeats. Gray lines between the DH1-v3 and DH1-v2 indicate collinear sequences. Red lines between the DH1-v3 and DH1-v2 indicate non-collinear sequences. **b**) Heat map of Hi-C contact information along the DH1-v3 chromosomes. Pixel colors represent different normalized counts of Hi-C links between 30-kb non-overlapping windows for all 9 chromosomes (Chr) on a logarithmic scale. Green lines represent the boundaries of individual contigs. Numbers in green represent the extra sequence in Mb that was new assembled compared to DH1 v2 assembly, into each chromosome.

**Extended Data Fig. 3:**
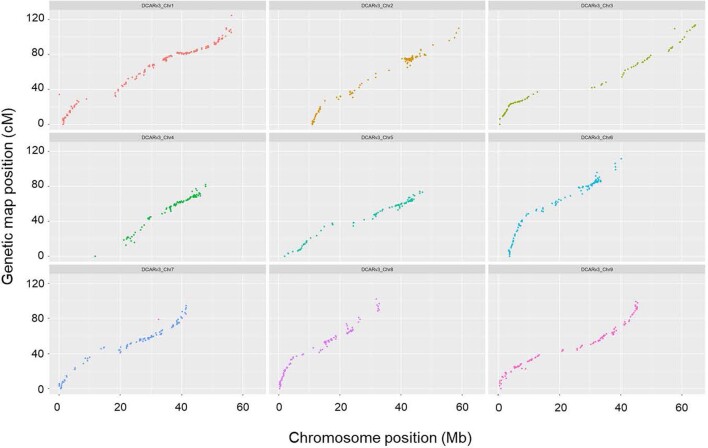
DH1 v3 assembly quality verification using a carrot linkage map. Comparison of the genetic map of population 3242^1^ to the physical map of the DH1 v3 genome assembly.

**Extended Data Fig. 4:**
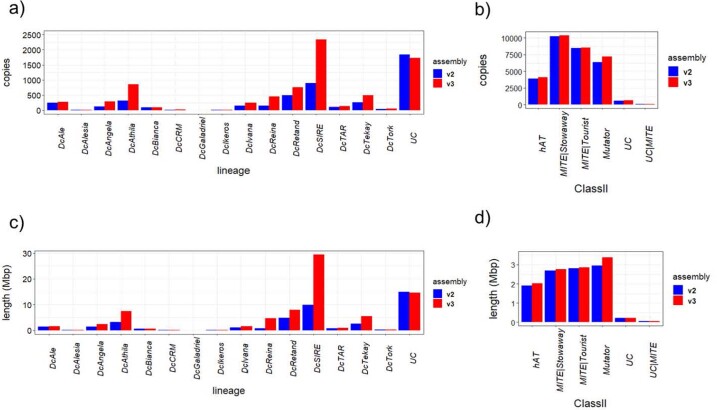
Comparison of mobile elements annotated in the DH1 v2 and v3 genome assemblies. Comparison of the number (**a**, **b**) and size (**c**, **d**) of full-length LTR retrotransposons (a, c) and TIR DNA transposons (b, d) between v2 (blue) and v3 (red) assemblies.

**Extended Data Fig. 5:**
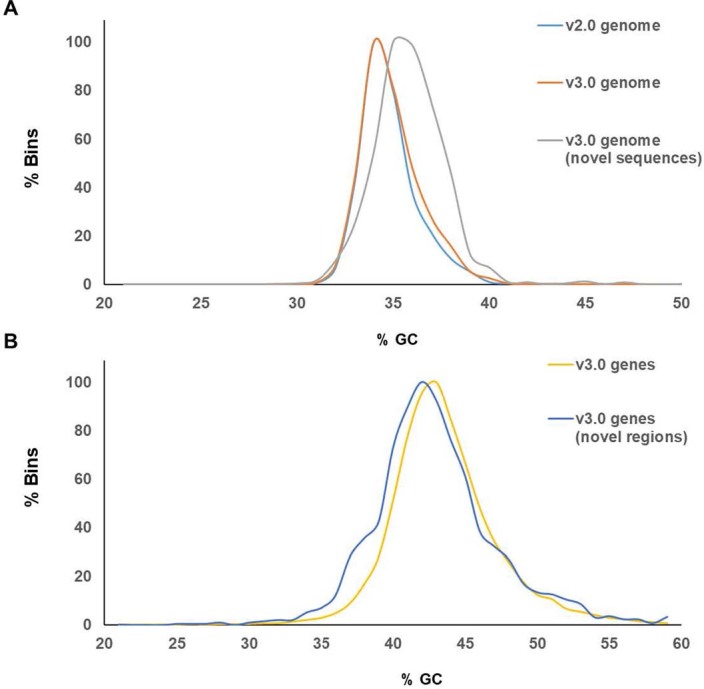
GC content estimated in the DH1 v2 and v3 genome assemblies. **a**) GC content in the v2, v3 genomes and in the newly assembled sequences in the v3 genome. **b**) GC content in the genes predicted in the v3 genome and in newly assembled sequences. Note, for each fraction of genome and genes evaluated in this analysis (for example v2 genome, v3 genes) the frequency of bins for each GC level (1% GC windows) was rescaled independently setting the minimum number of bins to 0 and maximum number of bins to 100, and plotted on the y axis. The calculation was carried out using mapminmax function implemented in Matlab.

**Extended Data Fig. 6:**
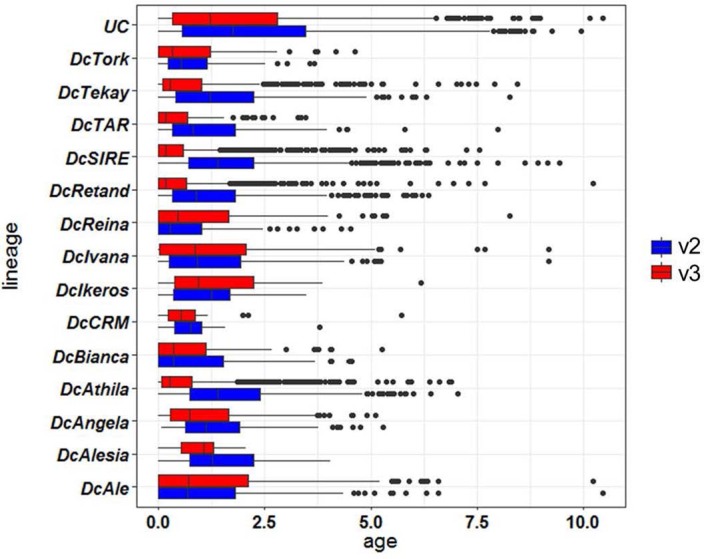
Transposable element age estimates. Age (Myr)distribution of full-length LTR retrotransposon lineages in DH1 v2 (blue) and v3 (red) carrot genome assemblies.*UCn*(v3)=1894, n(v2)= 2137; *DcTork* n(v3)= 52, n(v2)= 48; *DcTakay* n(v3)= 547, n(v2)= 383; *DcTAR* n(v3)= 139, n(v2)= 114; *DcSIRE* n(v3)= 2225, n(v2)= 1106; *DcRetand* n(v3)= 1184, n(v2)= 731; *DcReina* n(v3)= 157, n(v2)= 158; *DcIvana* n(v3)= 228, n(v2)= 213; *DcIkeros* n(v3)= 10, n(v2)= 8; *DcCRM* n(v3)= 22, n(v2)= 17; *DcBianca* n(v3)= 113, n(v2)= 103; *DcAthila* n(v3)= 918, n(v2)= 477; *DcAngela* n(v3)= 292, n(v2)= 222; *DcAlesia* n(v3)=8, n(v2)=7; *DcAle* n(v3)= 274, n(v2)= 252. The boxplot represents the 25^th^, 50^th^ and 75^th^ percentiles with the upper and lower whisker 1.5x the 75^th^ and 25^th^ percentiles, respectively.

**Extended Data Fig. 7:**
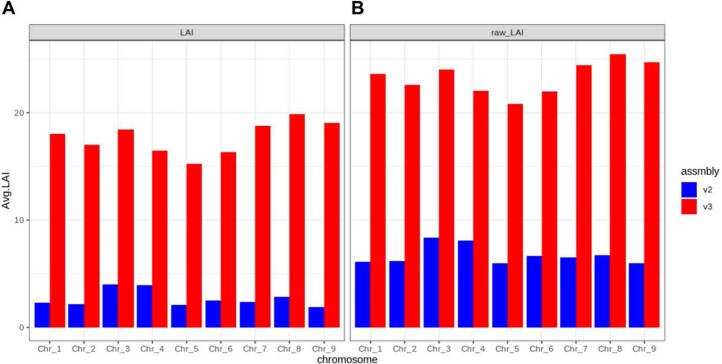
DH1 v2 and v3 assemblies’ quality verification using LTR Assembly Index (LAI). Normalized (**a**) and RAW (**b**) LAI lineages in DH1 v2 (blue) and v3 (red) carrot genome assemblies.

The new DH1 v.3.0 (hereafter DH1 v.3) assembly was developed using long-read (PacBio and Oxford Nanopore) and Illumina Hi-C sequence data (Supplementary Tables 1–3). The assembly spans 440.7 Mb, assembled into nine chromosomes that represent ~93% of the estimated genome size (473 Mb)^15^ (Table 1, Extended Data Fig. 1 and Supplementary Tables 4 and 5). Quality assessment for assembly contiguity, gene space coverage and sequence contaminations confirmed that the assembly reached high-quality standards (Supplementary Note, Extended Data Figs. 2 and 3, and Supplementary Tables 6–8). The overall N50 was 51 Mb, the contig N50 was over 6.0 Mb and the longest contig was over 28.0 Mb, covering much of the long arm of chromosome 4 (Fig. 1a and Extended Data Fig. 2). Compared with the DH1 v.2 assembly^15^, developed using Illumina short-read sequencing technology, DH1 v.3 has a >4-fold higher scaffold, a 193-fold higher contig N50 (Table 1) and about 21% newly anchored sequences. Also, a moderate number of sequence corrections were made around centromeric regions (Fig. 1a and Extended Data Fig. 2). As a result of these improvements, the DH1 v.3 assembly includes about 53.1 Mb (11.3%) more repetitive sequences (Supplementary Tables 9 and 10), largely represented by relatively young long terminal repeat (LTR) elements located in centromeric and pericentromeric regions (Fig. 1a, Supplementary Note and Extended Data Figs. 4–6) and a much higher LTR Assembly Index (22.88 versus 5.09) (Extended Data Fig. 7).

**Table 1:** Statistics and comparison of the carrot DH1 v.2 and v.3 genomes

	DH1 v.3			DH1 v.3 versus v.2			
	No.	Length (Mb)	Percentage (%)	No.	Percentage (%) or fold change	Length (Mb)	Percentage (%) or fold change
**Assembly feature**							
Sequences	9	440.7	93.2^a^	−4,817	−536-fold	+19.2	+4%
Contigs	563	440.6	93.1^a^	−30,375	−54.5-fold	+53.9	+12%
Min. contig length		0.014				+0.013	+27.8-fold
Max. sequence length		64.5				+13.1	+21%
Max. contig length		28.6				+28.6	+2,410-fold
Contig N50 length		6.1				+6	+193-fold
Scaffold N50 length		51.1				+38.4	+4.0-fold
Genome anchored		440.7				+78.7	+16.6%
Genome oriented		440.7				+87.6	+18.5%
**Genome annotation**							
Repetitive sequences		254.4	49.5^a^			+53.1	+11.3%
Gene models	36,216	42.8		+4,103	12%	+4.8	+12.7%
Genes in pseudomolecules	36,216	42.8	100	+5,392	15%	+5.9	+16.2%
Non-coding RNA	9,963			+43,448	+7.2-fold		
Resistance genes	4,279	3.8		+917	+27%	+0.6	+20.1%
Transcription factors	5,049	6.2		+1,037	+25%	+0.9	+17.5%

^a^Estimated considering the estimated genome size 473 Mb.

**Extended Data Fig. 8:**
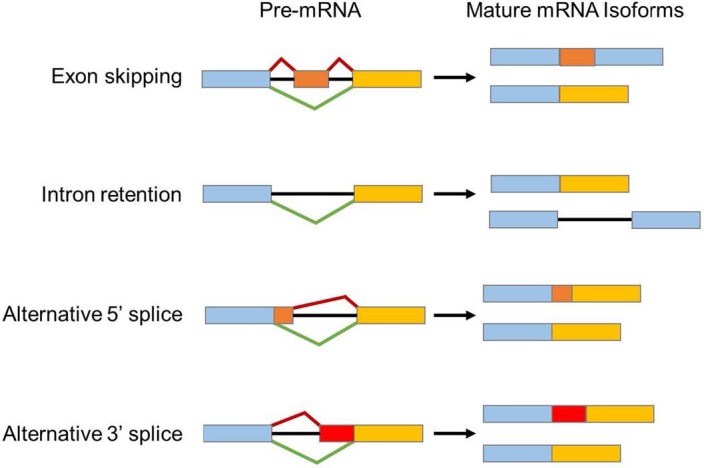
Gene isoforms. Schematic representation of the type of isoforms detected in carrot DH1 v3 genome.

**Extended Data Fig. 9:**
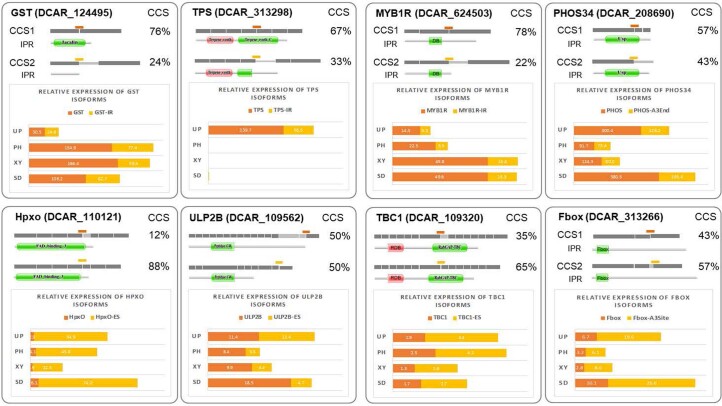
Validation of alternative splicing events in DH1 by qRT-PCR. Eight loci were analyzed, each expressing two isoforms generated from alternative splicing event. Glutathione S-transferase (GST), Terpene synthase (TPS), FAD-dependent hydroxylase (Hpxo) ubiquitin-like-specific protease 2B (ULP2B), MYB1R, Universal phosphorylated stress protein (PHOS34), TBC1 and Fbox. For each locus, the top scheme of the panel show the distribution of exons (dark grey rectangles), skipped exons (light grey rectangles) and retained introns (light grey lines) for both isoform, as well as the percentage of unique circular consensus sequence (CCS) detected in the IsoSeq libraries. The corresponding functional domain(s) predicted using Pfam (pfam.xfam.org) is displayed below each isoform. qRT-PCR reactions were designed to selectively amplify only one isoform. The position of the amplicon is indicated for each isoform by an orange or yellow line. Their level of expression was normalized to the ACTIN housekeeping gene using the ∆Ct method.

**Extended Data Fig. 10:**
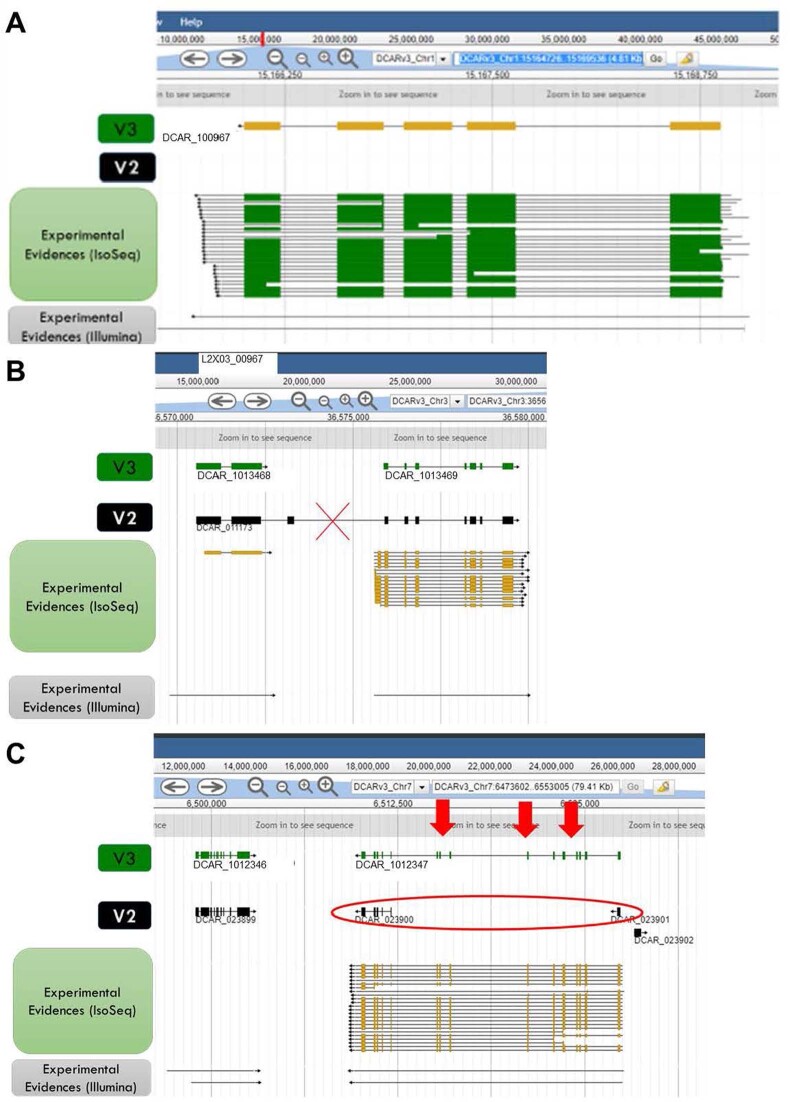
Comparison between the structure of the genes predicted in the v2 and v3 genome assemblies. **a**) Example of a gene predicted in v3 and not predicted in v2; **b**) example of a merged gene, predicted as one gene in v2 and two genes in v3; **c**) example of a split gene, predicted as one gene in v3 and two genes in v2. The quality of the predicted genes was supported by experimental evidence, including IsoSeq and Illumina transcriptome sequences.

In total, 36,211 protein-encoding genes were predicted in the DH1 v.3 genome (Table 1, Supplementary Tables 11 and 12 and Supplementary Note). Over 99.5% of the predicted genes had a match with Single Copy Ortholog, and 99.3% could be annotated (Supplementary Tables 13 and 14). Isoform analysis indicated that 15,723 predicted genes had more than one isoform, which can potentially change protein function by altering the conserved protein domains (Supplementary Note, Supplementary Tables 15 and 16, and Extended Data Figs. 8 and 9). In v.3, 4,103 additional genes were predicted compared with v.2, of which 3,084 were located in newly assembled sequences (Fig. 1a and Supplementary Table 17) and 98.2% were expressed, confirming the reliability of these predictions. Genes located in new regions were particularly enriched for gene families involved in electron transport (for example, *CB5-B*, *PSBO-2* and *CICDH*)^19–21^ and functioning in highly conserved processes such as photosynthesis or regulation of redox homeostasis (Supplementary Table 18). Comparing the alignments of genes in DH1 v.3 and DH1 v.2, 19,353 gene models had an identical start and end position, and 16,858 genes (48%) either were new in DH1 v.3 or had a different start or end position (Supplementary Table 19). IsoSeq reads confirmed the correctness of the new gene predictions (Extended Data Fig. 10).

To exemplify the improvement of the DH1 v.3 assembly, we reanalysed a region on chromosome 3 encompassing a MYB-TF named *DcMYB6* (DCAR_000385) that regulates anthocyanin accumulation in carrot root^22^ and that, in the v.2 assembly, was assembled into a short contig and not anchored to the chromosome sequences. In DH1 v.3, the region spanning *DcMYB6* was fully assembled into chromosome 3, and the regions flanking it were found to be composed of repetitive DNA carrying insertions of full-length *DcReina* and *DcAthila* nested into an older copy of *DcRetand* (Fig. 1b). The presence of nested LTRs from younger lineages that attained high copy numbers in the carrot genome (Fig. 1b) made it intractable to assemble this contig into chromosome 3 using the DH1 v.2 short-read assembly strategy and is now fully resolved using the longer read data in v.3. As a result, *DcMYB6* could be associated with a putative anthocyanin quantitative trait locus (QTL)^23^. In addition, the improved annotation method for the DH1 v.3 genome captured predictions for 1,037 new transcription factors and 917 new resistance genes (Fig. 1a, Table 1 and Supplementary Tables 20–23).

Overall, the characterization of the DH1 v.3 genome highlighted previously unknown features of the carrot genome, as well as the advantages that a higher-quality genome annotation can provide for the identification and characterization of biologically and economically relevant genes.

### Carrot population structure and phylogeny

A total of 630 carrot accessions, including wild carrots (*n* = 95), cultivars and landraces (*n* = 533), and outgroups (*n* = 2, *D. syrticus* and *D. sahariensis*), were resequenced to investigate carrot population dynamics, clustering, gene flow and demographic history (Supplementary Tables 24–26). These accessions were chosen to represent diverse geographic origins and breeding histories and to capture the extensive variation in traits associated with domestication and improvement, such as root colour, shapes, annual/biennial flowering and presence/absence of lateral branching. Resequencing resulted in the identification of 25,375,112 single nucleotide polymorphisms (SNPs), with 1,599,287 located within coding regions.

**Fig. 2:**
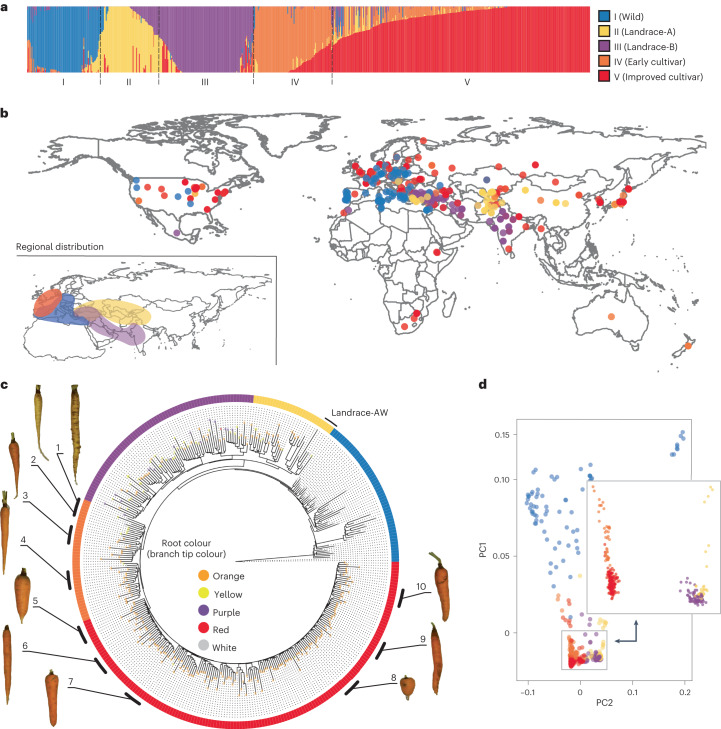
Population clustering of carrot germplasm. **a**, Population structure of 630 carrot accessions. The bar plot represents the percentage of membership (*q*) for each group identified at *K* = 5. The colour designations for each population (I–V) illustrated in **a** are used to represent accessions in all the other panels (**b**–**d**). **b**, Geographic distribution of the accessions according to the greatest proportion of ancestry at *K* = 5. The inset represents the distribution at the regional level of accessions grouped as Early cultivar, Landrace-A, Landrace-B and Wild populations that were located in more defined geographic regions. The Improved cultivars were spread across the world and are not represented in this inset. **c**, Neighbour-joining phylogenetic tree of 353 samples with <10% admixture proportions (low-admixture set). The consensus tree resulted from a bootstrap test (1,000 replicates). The branch tip colours represent the root colour phenotypes, and the outer ring corresponds to the population identity of each sample. The tree was rooted using *D. syrticus* as the outgroup. The numbers next to each carrot represent the following carrot cultivars/market types: (1) ‘Yellow Belgian’, (2) ‘Early Half-Long Horn’ (Yellow), (3) ‘Early Half-Long Horn’ (Orange), (4) ‘Long Orange’, (5) ‘Chantenay’, (6) ‘Altringham’, (7) ‘Amsterdam’, (8) ‘Oxheart’, (9) ‘Nantes’ and (10) ‘Amsterdam Forcing’. **d**, PCA of accessions (*n* = 630). PC1 and PC2 account for 4.0% and 2.8% of the total variation, respectively.

Population structure was inferred using a randomly sampled set of 168,410 linkage disequilibrium (LD)-pruned SNPs. Clustering analysis identified the strongest support for *K* = 5 populations (Fig. 2a and Supplementary Table 26). Population I, which includes wild carrots from Africa, Asia, Europe, and North and South America, is referred to as the Wild population (Fig. 2a,b and Supplementary Fig. 1a,b). Populations II and III, referred to as Landrace-A and Landrace-B, respectively, represent the Eastern carrots and include accessions with somewhat undomesticated phenotypes such as non-uniformity within accessions or non-smooth roots. However, these populations also had clearly domesticated characteristics including reduced lateral root branching and the presence of anthocyanin or carotenoid pigmentation (Supplementary Fig. 1b). Accessions belonging to Landrace-A represent carrots from central and eastern Asia, while Landrace-B accessions represent carrots from western and southern Asia in the geographic area spanning from Turkey to India (Fig. 2b). In addition to carrot accessions with landrace phenotypes, the Landrace-A population included 15 wild accessions (hereafter Landrace-AW) (Supplementary Fig. 1b), perhaps derived from intercrosses with cultivated carrots, all from central Asia, where farmers’ seed production is often very close to wild carrot populations (Supplementary Fig. 1b). Further analysis of gene flow and demographic history (Supplementary Note and Supplementary Figs. 2 and 3) indicated that Landrace-AW probably represents a feral lineage of carrot that escaped from cultivation and re-established in the wild. Two additional populations (IV and V), named the Early cultivar and the Improved cultivar, represent Western carrots, which originated mostly in Europe and North America (Fig. 2a,b). Accessions belonging to these populations exhibit morphological phenotypes similar to modern carrot cultivars, such as uniform root shape and the accumulation of high amounts of orange carotenoid pigments (Supplementary Fig. 1b). Early cultivars represent the ‘Horn’ and ‘Long Orange’ carrot market types that were the founders of Western orange carrot. Improved cultivars represent orange market-type cultivars such as ‘Nantes’, ‘Amsterdam Forcing’, ‘Chantenay’ and ‘Danver’, which were developed between the eighteenth and nineteenth centuries in response to the increasing demand for orange carrots in Europe and globally. Over 261 (41%) accessions harbour >10% alleles derived from more than two populations (Supplementary Table 26), indicating a high level of inter-population admixture that reflects the outcrossing nature of carrot^24^. To avoid bias due to potential ancestry admixture, downstream analyses were also conducted using low-admixture samples (ancestry coefficient >0.9 for a given reference population).

**Fig. 3:**
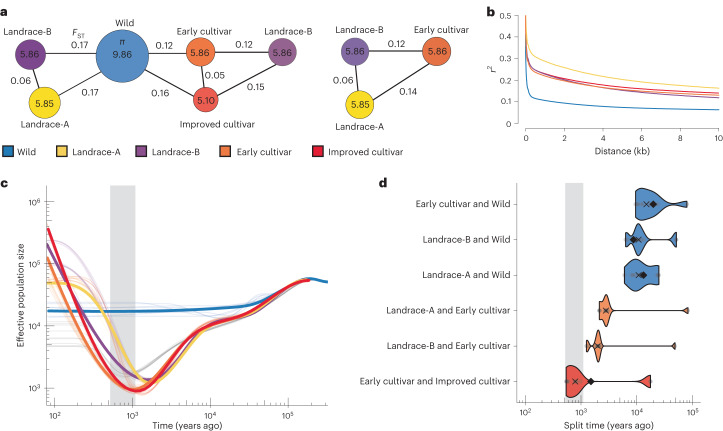
Genetic diversity and demographic analysis of carrot germplasm. **a**, Nucleotide diversity and *F*_ST_ values between carrot populations using the low-admixture dataset (*n* = 353). The numbers in the circles represent *π* values; the numbers outside the circles represent *F*_ST_ values. The *π* values for each population represent the 10^−3^ decimal scale (for example, 0.00986 for Wild). **b**, LD decay across the five carrot populations (low-admixture set). **c**, Effective population size trajectories for the carrot populations estimated using SMC++, with the estimate uncertainty displayed as lighter lines for ten bootstrap replicates. Both the *x* and *y* axes are on a log_10_ scale. **d**, Inferred divergence times for pairs of carrot populations. Point estimates based on all data are displayed as solid black diamonds, with uncertainty displayed for ten bootstrapped replicates (the lighter points and violin plots, with a cross to denote the median of the bootstrapped estimates). The colours indicate the population that is being compared against. The *y* axis is on a log_10_ scale. The grey boxes in **c**,**d** indicate the hypothesized window of carrot domestication from 522 to 1,100 years ago.

Phylogenetic analysis and principal component analysis (PCA) support the separation of five populations (Fig. 2c,d and Supplementary Figs. 4–6). Wild and cultivated accessions formed two distinct clades, except for five wild accessions, Landrace-AW (Fig. 2c). Landrace-A and Landrace-B populations were distinct from accessions belonging to the Early cultivar and Improved cultivar populations (Fig. 2c,d). These results suggest that the Landrace-A and Landrace-B populations share a common origin (Fig. 2c), which was reinforced by the low *F*_ST_ estimate (*F*_ST_ = 0.06) between Landrace-A and Landrace-B, indicating a low amount of differentiation between these two populations (Fig. 3a and Supplementary Table 27). Gene flow was detected between these two populations (Supplementary Table 28) and probably contributed to this low differentiation. The Improved cultivar and Early cultivar populations clustered into a separate sister clade and formed two distinct subclades, with the Early cultivar clade being ancestral to the Improved cultivar clade. Interestingly, a group of yellow carrots from the Netherlands and Poland clustered at the base of all the Early and Improved cultivars, which supports the hypothesis that the ‘Long Orange’ and ‘Horn’ types were selected in Europe from yellow carrots^5^ and that these populations formed the basis of Western and modern orange carrot varieties. The topology of the phylogenetic tree suggests that Western carrots are not directly descended from Eastern carrots but share a common ancestor with wild carrots, possibly due to hybridization between these populations. Supporting this hypothesis, evidence of gene flow between Early cultivars and Wild populations was detected using *f*_4_-statistics and TreeMix^25^ analysis (Supplementary Table 28 and Supplementary Fig. 2), with a TreeMix migration edge indicating that gene flow occurred from Early cultivars into Wild accessions. This result was also reinforced by *F*_ST_ estimates, which indicated that, among cultivated and landrace accessions, Early cultivars have the least amount of differentiation (*F*_ST_ = 0.12) from the Wild population (Fig. 3a and Supplementary Table 27). Relationships among carrot populations were further clarified using outgroup *f*_3_-statistics, represented as *f*_3_(reference population, test population; outgroup), using wild samples from *D. carota* subspecies as the outgroup population (subsp. *gummifer*, *maximus carota* and *maritimus carota*). The results support the relationships inferred from the phylogeny, with the Wild accessions having diverged from a common ancestor first, followed by the Landrace-A and Landrace-B populations, and lastly the Early and Improved cultivar populations (Supplementary Fig. 7).

### Carrot genetic diversity

Analysis of genetic diversity within the low-admixture set indicated that nucleotide diversity was substantially higher for wild carrots (*π* = 9.86 × 10^−3^) than for landraces (*π* = 5.85 × 10^−3^ to 5.86 × 10^−3^) and cultivars (*π* = 5.81 × 10^−3^ to 5.86 × 10^−3^) (Fig. 3a and Supplementary Table 29). Similar results were obtained using the full set (Supplementary Table 29). Among cultivated accessions, nucleotide diversity was lowest in the Improved cultivars (*π* = 5.81 × 10^−3^), which reflects their status as highly selected populations (Fig. 3a). Additionally, a survey of the half-life of LD decay occurred at 57 nucleotides (nt) in the Wild population, while the Early cultivar and Improved cultivar populations exhibited an LD decay half-life of 315 nt and 348 nt, respectively (Fig. 3b). The slower rate of LD decay in cultivated carrot populations suggests a substantial decrease in genetic diversity following domestication and improvement.

### Carrot demographic history

To investigate the demographic history of each carrot population, SMC++^26^ was used to infer population size histories (Fig. 3c). Individuals used in this analysis were restricted to samples with low admixture. Effective population size (*N*_e_) trajectories support a shared bottleneck followed by recent expansion. We observed equivalent or increased *N*_e_ in modern populations relative to ancestral *N*_e_ in the Landrace-A, Landrace-B, Early cultivar and Improved cultivar populations, with minima occurring at ~1,360, 1,206, 953 and 895 years ago, respectively. This result is consistent with historical documents, which place the period of carrot domestication in central Asia (Landrace-A region) between the ninth and tenth centuries, approximately 1,200 years before present, and the selection and improvement of Western orange carrots (Early and Improved cultivars) in the sixteenth and seventeenth centuries, between 500 and 600 years before present^5,27^ (Fig. 3c). No corresponding bottleneck was observed in the Wild population, further supporting the idea that the observed reduction in *N*_e_ probably coincides with the period of domestication in the landrace and cultivar populations.

To estimate divergence between populations, SMC++ uses a ‘clean split’ model, which assumes there is no gene flow following a split between populations. When post-split gene flow occurs, the model is expected to underestimate divergence times^28^. When estimating divergence among carrot populations, the deepest splits were observed for the landrace and cultivated populations compared with the Wild population, with median estimates of divergence ranging from ~10,804 to 14,970 years ago (Fig. 3d). Subsequent divergence times between Early cultivars and Landrace-A and Early cultivars and Landrace-B were estimated at 2,803, and 1,998 years ago (median), respectively (Fig. 3d). Bootstrapped estimates support the most recent split occurring between the Early and Improved cultivar populations (median of ~788 years ago).

### Selective sweeps for carrot domestication and improvement

**Fig. 4:**
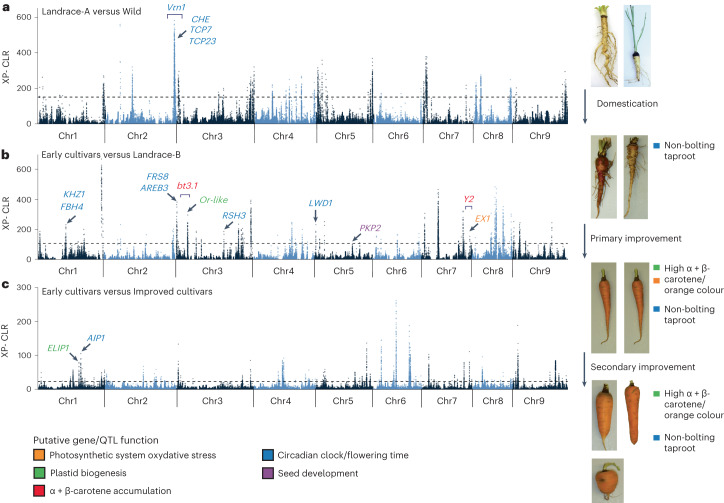
Selective sweep analysis across carrot populations. **a**, Selective sweeps and candidate genes contrasting the Wild and Landrace-B populations (domestication sweeps). **b**, Selective sweeps and candidate genes contrasting the Landrace-B and Early cultivar populations (improvement sweeps). **c**, Selective sweeps and candidate genes contrasting the Early cultivar and Improved cultivar populations (improvement sweeps). Previously known loci are indicated with square horizontal brackets. The carrot pictures and short descriptions presented on the right side of each panel represent a possible change in plant characteristics that occurred during domestication and cultivar improvement. Each selective sweep panel on the right side (**a**–**c**) is a representative comparison among the populations that correspond to that specific step (domestication, selection or improvement).

To identify selective sweeps, pairwise scans were performed between the five populations. Selective sweeps identified between the Wild population and Landrace-A and Landrace-B were considered as those involved in domestication, while those between Landraces A and B and the Early and Improved cultivars were involved in improvement (Supplementary Table 30). In total, 18 distinct genomic regions were identified as selective sweeps (Fig. 4 and Supplementary Table 30). Analysis for genes underlying the selective sweeps identified several enriched gene families, including those related to photoperiodism and circadian clock regulation, control of flower development, photosynthesis, and regulation of isoprenoid metabolic processes (Supplementary Note and Supplementary Table 31).

Delayed flowering is a critical trait for domestication because the taproot becomes fibrous and inedible once flowering occurs. Interestingly, within a selective sweep associated with domestication, genes involved in circadian clock regulation and flowering time (including homologues of *CCA1 HIKING EXPEDITION* (*CHE*)^29^, *TCP23* (ref. ^30^) and *TCP7* (ref. ^31^)) were enriched (Fig. 4a and Supplementary Tables 31 and 32). This region overlaps with the region spanning the vernalization (*Vrn1*) locus previously mapped in carrot (Supplementary Table 32)^32^.

Multiple selective sweeps associated with improvement also harboured homologue genes involved in flowering time regulation (*KHZ1*, *FBH4*, *AREB3*, *LWD1* and *CIB4*)^33–37^. As domesticated carrot spread into multiple geographic regions, selection for genes involved in flowering time regulation continued to play a critical role in adaptation to multiple environments (Fig. 4b,c and Supplementary Table 32).

The increasing accumulation of carotenoids in the taproot has been a major focus of modern carrot breeding. The QTL *Bt.3.1* (ref. ^17^) on chromosome 3 co-localized with the primary improvement sweep identified on chromosome 3 that harbours the *Or-like* gene (Fig. 4b and Supplementary Table 32). *Or* genes control chromoplast biogenesis and enhance the preferential accumulation of β-carotene^38–40^. Another improvement selective sweep harboured the gene *ELIP1*, which is known to interact with *Or* to regulate chloroplast biogenesis^41^. The *Y2* QTL^16^ on chromosome 7 overlaps with a selective sweep that harbours DCAR_730022, a gene that was identified here (see below) as a new candidate gene controlling this QTL. DCAR_730022 shares homology to *EXECUTER1*, which mediates the response to singlet oxygen within the chloroplast^42,43^. Breeding for high-carotenoid phenotypes may have indirectly led to the selection of genes involved in plastid biogenesis and the cross-talk between the photosynthetic system and carotenoids accumulating in the carrot root.

### Genome-wide association analysis for carotenoids

**Fig. 5:**
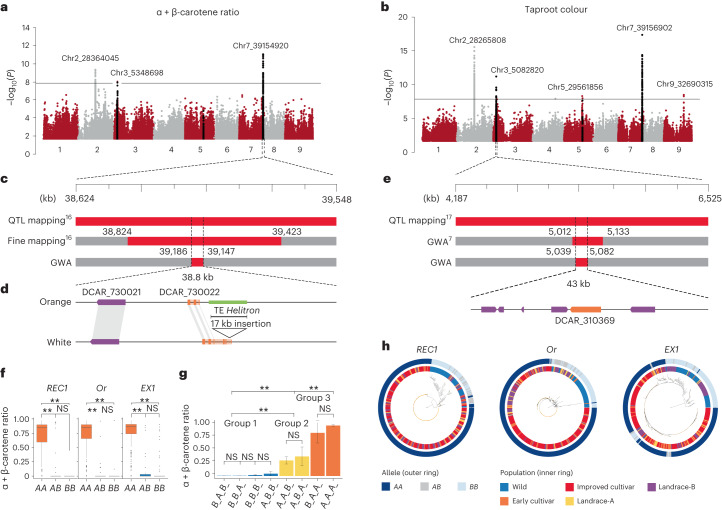
Candidate genes for taproot colour and carotenoid concentration identified by association mapping. **a**, Manhattan plot of GWA analysis for the ratio of α + β-carotene relative to total carotenoids. **b**, Manhattan plot of GWA analysis for taproot colour. **c**, Overlap of the significant locus identified on chromosome 7 with the previously mapped *Y2* locus^16^ and a 38.8-kb region containing the top 30% of markers most associated with carotenoid accumulation. **d**, Comparative analysis of the region spanning the *Y2* locus in DH1 and Lunar-White, a white accession with the wild *Y2* allele. The *Y2* candidate gene (DCAR_730022) is highlighted in orange. The structure of DCAR_730022 in DH1 and Lunar-White and a 17-kb insertion detected in the DH1 *Y2* gene are illustrated. **e**, Overlap of the significant locus identified on chromosome 3 with the previously mapped *Or* locus^7,17^. A region of 43 kb was identified here as the most significantly associated with *Or* that harbours the gene controlling this locus. Predicted genes within the locus identified on chromosome 3 are illustrated, and the *Or-like* gene (DCAR_310369) is highlighted in orange. **f**, The effects of the three different alleles (*AA*, *AB* and *BB*) at the *REC1*, *Or* and *EX1* candidate genes on the ratio of α + β-carotene concentration to total carotenoid content. Statistical analysis was performed using the *F* statistical test. The box plots represent the 25th, 50th and 75th percentiles, and the upper and lower whiskers represent 1.5× the 75th and 25th percentiles, respectively. For *REC1*, *n*(CC) = 14, *n*(AC) = 32 and *n*(AA) = 360; for *Or*, *n*(GG) = 28, *n*(AG) = 27 and *n*(AA) = 351; and for *EX1*, *n*(TT) = 59, *n*(AT) = 27 and *n*(TT) = 320. **g**, *REC1*, *Or* and *EX1* allelic interaction analysis. The data are presented as the mean plus or minus the s.e.m. For *B_A_B_*, *n* = 11; for *B_B_A_*, *n* = 11; for *B_B_B_*, *n* = 12; for *A_B_B_*, *n* = 16; for *A_A_B_*, *n* = 19; for *A_B_A_*, *n* = 20; for *B_A_A_*, *n* = 23; and for *A_A_A_*, *n* = 294. **h**, Neighbour-joining phylogenetic tree of the SNPs identified at the *REC1*, *Or* and *EX1* genes across the low-admixture set. The consensus trees were constructed from 1,000 bootstrap replicates. The outer ring corresponds to the allele detected for each sample (*AA*, *AB* or *BB*). The inner ring represents the population identity of each sample. The asterisks in **f**,**g** indicate allelic groups that were significantly different (*P* < 0.01); NS indicates allelic groups that were not significantly different.

Carotenoid accumulation was investigated using genome-wide association (GWA) analyses of the visual taproot phenotypes for 601 accessions and with the relative carotenoid content of 435 accessions (Supplementary Note and Supplementary Table 33). The most significant loci were mapped in chromosomes 2, 3 and 7 and were associated with taproot colour and the ratios of α + β-carotene and lutein to total carotenoids, while four weaker loci were identified in chromosomes 5 and 9 and were associated only with root colour (Fig. 5a,b and Supplementary Table 34).

The most significant locus detected on chromosome 7 overlapped with the fine-mapped *Y2* QTL region that controls the orange phenotype in carrot (Fig. 5c)^16^. The region spanning the top 30% of the most significant SNPs included two candidate genes, DCAR_730021 and DCAR_730022 (Fig. 5d and Supplementary Tables 35 and 36). DCAR_730022 was downregulated in orange samples harbouring the recessive *Y2* allele and harboured SNPs with stronger associations (Supplementary Note and Supplementary Tables 37 and 38). Also, an insertion of a *Helitron* disrupting the DCAR_730022 coding sequence (CDS) was identified in DH1 and 97% of the orange accessions (Fig. 5d, Supplementary Note and Supplementary Table 39). Transcriptional interactome network analysis identified DCAR_730022 as a key link in the interaction between genes involved in ‘Photosystem PSII associated light-harvesting complex’, including ‘singlet oxygen response’ (^1^O_2_) along with isoprenoid biosynthetic pathways (Supplementary Tables 40 and 41 and Supplementary Fig. 8). In line with these results, DCAR_730022 shares partial homology to *EXECUTER1* (*EX1*), which is known to be involved in activating the enzymatic ^1^O_2_ stress response program in plants to repair photosystem II^43,44^. Interestingly, the non-enzymatic breakdown of β-carotene, a ^1^O_2_ scavenger, represents the alternative mechanism of reactive oxygen quenching in photosystem II^45,46^. Considering these results, it is plausible that a non-functional *EX1-like* gene in genotypes carrying the insertion, such as DH1, could cause the plant to maintain high levels of β-carotene biosynthesis to quench ^1^O_2_. This possible mechanism, its expression and the disruption of the CDS in orange samples (which is compatible with a recessive mutation like *Y2*) provide compelling evidence for pursuing functional validation of DCAR_730022 as the *Y2* candidate gene.

The significant associations mapped on chromosome 3 overlap with the previously identified *Or* locus (Fig. 5b and Supplementary Table 34)^7,17^. A survey of the region within 30% of the top-scoring SNPs yielded six genes (Fig. 5e and Supplementary Table 35). The gene DCAR_310369, orthologous to the *Arabidopsis* *Or-like* gene, was the only gene located within this region that has been associated with carotenoid accumulation in carrot and other species^7,39^ (Supplementary Table 36). Recent work in carrot demonstrated that knocking down the expression of DCAR_310369 in an orange carrot genotype resulted in yellow carrot^47^. Notably, this gene was not differentially expressed between yellow carrots carrying the dominant allele and orange carrots carrying the recessive allele (Supplementary Note and Supplementary Tables 42 and 43), suggesting that its function may be controlled at the protein level as reported in other plant systems^38,40^.

The locus mapped in chromosome 2 represents a new locus related to carotenoid accumulation in carrot (Fig. 5a,b and Supplementary Table 34). The region harbours 26 positional candidate genes and includes one gene, DCAR_206039, homologous to *Arabidopsis reduced chloroplast coverage 1* (*REC1*)^48^. A *REC1* orthologous gene in *Mimulus* (*RCP2*) directly affects carotenoid content^49,50^ (Supplementary Note and Supplementary Table 35).

### Carotenoid gene effects and interactions

Next, SNPs detected within *EX1-like* (DCAR_730022), *Or-like* (DCAR_310369) and *REC1-like* (DCAR_206039) were used to evaluate their effects and interactions in relation to the ratios of α + β-carotene content to total carotenoid content and visual orange phenotypes. Single marker effect analysis indicated that *Or-like*, *EX1-like* and *REC1-like* contribute to a significant (*P* < 0.001) increase of the α-carotene and β-carotene concentration. The results also indicated that the recessive alleles for all three genes (hereafter cultivated, *A*, *REC1_A*, *Or_A* and *EX1_A*; Fig. 5f) as opposed to the dominant wild alleles (hereafter wild, *B*, *REC1_B*, *Or_B* and *EX1_B*; Fig. 5f) condition carotenoid accumulation. The recessive genetic model for *EX1-like* fully agrees with previous studies performed in multiple mapping populations^14,16,17^. Also, for *Or-like*, the allele associated with the recessive model in this analysis (homozygous TT at position 551) corresponds to the allele coding for leucine (named *DcOR3*^*Leu*^), which has been proved to control the orange phenotype in carrot^47^. These observations confirm the robustness of the results presented here at the population level.

Two-way epistatic interactions exist between the three loci (*P* < 0.001) except *REC1* and *Or*. Also, a three-way interaction among all the alleles was significant (*P* < 0.05). On the basis of analysis of variance for allele interaction and the ratios of α + β-carotene to total carotenoids, the genotypes could be separated into three groups (*P* < 0.05) (Fig. 5g). Group 1 included genotypes that either harbour only one of the recessive alleles or are missing all of them (for example, *REC1_B/Or_B/EX1_B*). Among these genotypes, only one was orange, and the fraction of α + β-carotene was very low (<0.1%) or not detected (Fig. 5g). Group 2 included genotypes that harboured the *REC1* recessive allele and either the *Or* or *EX1* wild allele (for example, *A_A_B_*). Among these genotypes, 25% were orange (mostly pale orange), with a fraction of α + β-carotene that was significantly higher (average 0.24%) than group 1 (Supplementary Figs. 9 and 10). Group 3 included genotypes that harboured recessive alleles for all three genes or harboured *Or* and *EX1* recessive alleles (for example, *A_A_A_*). Among these genotypes, 96% were orange (nearly all dark orange), and the fraction of α + β-carotene in these genotypes was the highest (average 0.78%) (Supplementary Figs. 9 and 10). Overall, these results demonstrate that the recessive alleles at both *Y2* and *Or* are strictly needed to select orange carrot with high concentrations of α + β-carotene, and a recessive allele at the *REC1* locus contributes to reaching the highest concentrations of α + β-carotene.

To gain some preliminary insight into the selection process of these three genes, we carried out phylogenetic analysis with SNPs spanning the *REC1*, *Or* and *EX1* genes (CDS) from the low-admixture set. The results indicated a clear separation of genotypes that harbour the recessive alleles, found in cultivated accessions, from those that harbour the dominant wild alleles (Fig. 5h). The clades including the cultivated alleles included nearly all orange genotypes as well as a limited number of non-orange genotypes (for example, purple). Relative to the five populations, for all three genes, the phylogenies clustered the same populations of domesticated carrot (Landrace-A, Landrace-B, and the Early and Improved cultivars) together. These results indicate that the origin of the orange cultivated alleles for all three genes is monophyletic; each gene was probably selected once and rapidly fixed as soon as the orange phenotype was selected. This assertion is also supported by the shared genetic bottleneck identified in the demographic analysis.

## Discussion

Historical documents and previous studies indicate that carrot germplasm can be separated into three major groups (Eastern, Western and Wild carrots) and suggest that Eastern carrots were domesticated in central Asia^6,15^ and formed the basis of Western carrots^5,6,27,51^. However, the demographic events that characterized carrot domestication and improvement have not been assessed to support this hypothesis. In this study, an improved carrot genome assembly and resequencing of 630 diverse carrot accessions that represent the global distribution of carrot germplasm were used to reconstruct a detailed picture of carrot domestication and improvement, as well as the consequences of these selection processes for the genetic makeup of this important crop.

The separation between Wild, Eastern and Western populations was confirmed. Eastern and Western carrots were further separated into subpopulations, named here Landrace-A and Landrace-B for Eastern carrots, and Early cultivars and Improved cultivars for Western carrots. Phylogenetic analysis indicated that the progenitor of Western carrots shared its ancestry with Eastern and Wild carrots, in contrast to the standing hypothesis of Eastern carrots as the progenitor of Western carrots. However, gene flow analysis indicated that the signature of wild ancestry detected in the Early cultivars was confounded by hybridization between Early cultivars and the Wild population, particularly due to the movement of alleles from cultivated to wild populations. Considering that carrot is an outcrossing species and that wild carrot is often found in areas of cultivated carrot seed production, gene flow between wild and cultivated carrots can easily occur^52^. On the basis of these results and observations, this study still lends support to the hypothesis that Eastern carrots are the progenitor of Western carrots. However, we cannot exclude the possibility that Western carrots originated from an unsampled or extinct population. Furthermore, given that Landrace-A and Landrace-B represent sister populations and have evidence of gene flow between them, the origin of Western carrots cannot be specifically traced to one of the two populations.

Population divergence estimates strongly support the documented chronological history of carrot domestication and improvement. Demographic analysis indicates that recent population expansion in Eastern carrots began ~1,300 years ago, with the more recent expansion of orange Western carrot cultivars estimated to have begun about 800 years ago. These estimates closely match existing timelines from historical records, which indicate that Eastern carrots were documented in central Asia between 1,100 and 1,500 years ago^5,27,51,53^. On the basis of historical records and our demographic analysis, carrot domestication can be placed between the sixth and tenth centuries, during the Early Middle Ages. The distribution of the Landrace-A and Landrace-B populations coincides with the separation between western-southern Asia (Turkey, Iran and India) and central-eastern Asia (Afghanistan, Tajikistan, Uzbekistan, Pakistan, China and Japan), and overlaps with Asia Minor and central Asia, respectively. Divergence time estimates support the separation of the Landrace-A population from wild carrots earlier than Landrace-B, suggesting that the domestication of central Asian carrots pre-dated the spread of carrot in Asia Minor.

The more recent population expansion detected for the Early and Improved cultivar samples began about 800–900 years ago. This estimate matches the selection and documented spread of Western orange carrot between the sixteenth and eighteenth centuries^53^. Historical records also indicate that between the twelfth and fifteenth centuries, yellow and purple carrot were used in Spain, Italy, France, Germany, England and the Netherlands^5^. However, yellow carrots became more popular in Europe and probably established the basis of Western carrot^5,53^. This chronological reconstruction based on molecular and historical data was corroborated by the phylogenetic analysis, which placed a number of Western yellow carrots as the founders of the Early cultivars at the base of the market types ‘Horn’ and ‘Long Orange’, which are known to be the founders of the orange carrot types^5^. Clustering of ‘Yellow Belgian’ and other yellow carrots from the Netherlands as the progenitor of all Western orange carrots provides strong support for one of the most debated hypotheses proposed in 1963^5^, which suggests a Dutch (or perhaps Belgian) origin of Western orange carrots that were selected from yellow domesticated carrots.

As demonstrated by our phylogenetic analysis, Early cultivars were the founders of the Improved cultivars. These results coincide with historical records indicating that, after the selection of the orange phenotype occurred in Europe, orange carrots became very popular, and new cultivars with reduced high intra-cultivar uniformity and with specific root shapes or market types (for example, ‘Nantes’, ‘Amsterdam Forcing’ and ‘Chantenay’) were developed during the seventeenth and eighteenth centuries to meet the growing global demand^5,27,54^.

Previous studies have indicated that after domestication cultivated carrot experienced limited or no reduction of genetic diversity^6,7,15^. In contrast, our estimates of nucleotide diversity and effective population size suggest that a progressive reduction of genetic diversity accompanied carrot domestication and improvement. The higher SNP density and sequences captured (especially intergenic regions) in the DH1 v.3 genome assembly probably contributed to resolving controversial results from previous studies. As demonstrated in this study, strong selection pressure was detected for domestication traits such as vernalization and improvement traits such as orange roots. Given that these phenotypes are under the control of recessive alleles^15,16,32^, they were probably used as a visual tool for carrot breeders to keep cultivated carrot relatively free from outcross contamination by wild species. This process probably contributed to the reduction of genetic diversity in cultivated carrot.

Selective sweep analysis identified selection and/or fixation for genes related to flowering and high carotenoid pigmentation. These results are consistent with our knowledge about the traits selected during carrot domestication and improvement and support the role of conscious and/or unconscious selection by farmers and breeders on traits of economic value. For instance, delayed flowering in carrot is strictly needed to produce a nutrient-rich edible root^55^. The finding that genes controlling flowering time were enriched within the selective sweep regions demonstrates that this trait played an important role during the initial domestication and improvement of carrot and probably enabled their adaptation to and cultivation in different regions of the world. The overlap of a major domestication selective sweep with *Vrn*^32^, a vernalization locus previously mapped in chromosome 2, provides strong support for these results.

The GWA and selective sweep results suggest that the high-carotenoid phenotype in modern carrot cultivars is the result of a complex interaction between the response to light perception, plastid biogenesis and development, and carotenoid biosynthesis. The importance of previously mapped loci (*Y2* and *Or*) in regulating orange carotenoid accumulation in carrot roots was confirmed, and a new candidate locus (named here *REC1*) was mapped on chromosome 2. The previously characterized *Or-like* gene was confirmed to be the gene controlling the *Or* locus^7,17^, and two new candidate genes, *EX1-like* and *REC1-like*, were identified for the *Y2* and *REC* loci, respectively. Although the role of *EX1-like* and *REC1-like* will need to be verified through functional analysis, the rapid LD decay detected in carrot populations provides high resolution for gene mapping and support for their candidacy. For instance, the recessive genetic model established for *EX1* at the *Y2* locus matches the results from previous studies^7,16,17^. Other evidence supporting the role of these genes in controlling carotenoid accumulation includes gene expression analysis (*EX1*), causal mutation analysis (*Or* and *EX1*) and functional annotation indicating that all three genes belong to gene families that regulate or mediate the interaction between the carotenoid biosynthetic pathway, the photosynthetic systems and chloroplast biogenesis.

The large-scale population genomic analysis performed here provides an example investigation of the selection process underlying the orange phenotype at the gene level. The results indicate that the recessive cultivated alleles at all three genes—*REC1*, *Or* and *EX1*—were essential to select the orange phenotype, and each cultivated allele was selected once and rapidly fixed. *Or* and *EX1* were essential to reach the highest fraction of α + β-carotene, while *EX1* or *Or* in combination with *REC1* led to the accumulation of a low-medium fraction of α + β-carotene that is mostly associated with a pale-orange root phenotype. As these genes are located on different chromosomes, carrots with different *REC1*, *Or* and *EX1* cultivated allele combinations may have been developed independently. As a result, multiple orange phenotypes may have been developed in parallel. Carrots with a lower fraction of α + β-carotene and a pale-orange phenotype probably pre-dated or paralleled the selection of the dark-orange phenotype. Interestingly, this hypothesis is supported by historical documents indicating that in the seventeenth century, both types of orange carrots (pale and dark orange) were clearly identified^27^. Due to their reciprocal epistatic effect on the orange colour, once this trait was selected, the orange alleles were fixed.

This study elucidated the demographic history of carrot domestication and breeding and demonstrated that selection for the *REC*, *Y2* and *Or* QTLs established the basis for modern-day orange carrot. The new DH1 v.3 genome provides a valuable resource to advance genetic mapping, comparative genomics and gene cloning studies. Building on these findings, future work based on long-read sequencing technology and phased genomes can further trace the ancestry of the *REC1*, *Or* and *EX1* genes. This foundational work will enable further studies on the genetic mechanisms regulating carotene accumulation in carrot, with potential applications to other crops.

## Methods

### Sequencing and de novo assembly

For de novo assembly of the DH1 genome (doubled haploid orange Nantes type carrot, NCBI Biosample SAMN03216637), sequencing was performed with Pacific Biosciences (PacBio), Oxford Nanopore and Hi-C sequencing technologies (see the Supplementary Note and Supplementary Tables 1–3 for more details). A detailed description of the genome assembly method is described in the Supplementary Note and Supplementary Table 45 and illustrated in Extended Data Fig. 1. A list of the software and parameters used has also been made available through GitHub (https://github.com/dsenalik/Carrot_Genome_DH1_v3).

### Assembly quality verification

A comprehensive analysis was carried out to evaluate the quality of the final carrot DH1 v.3 genome assembly. Fastq-Screen (v.0.4.14)^56^ and GC content distribution estimates were used to assess the presence of sequence contaminations (see the Supplementary Note for more details).

The correctness of the assembled sequences was evaluated by estimating the mapping distance between a set of 4,717 Bacterial Artificial Chromosome End Sequencing (BES) that unambiguously aligned with both ends to the DH1 v.3 genome assembly and that were not used during the assembly process. The fraction of Paired-end (PE) data that aligned within the expected library insert size should reflect the fraction of assembled sequences that are consistently contiguous and correctly assembled. Also, a linkage map that included 3,242 markers^57^ not used for genome assembly was used to independently verify the order of the sequences. Marker sequences were mapped using BWA mem^58^ (see the Supplementary Note for the parameter and filtering settings).

Gene space coverage was assessed using carrot expressed sequence tags^59^, DH1 IsoSeq full-length transcripts generated in this study and 20 sets of publicly available DH1 Illumina transcriptome data. Expressed sequence tags were mapped using BWA mem, StringTie (v.1.3.5)^60^ was used to map the Illumina transcriptome data and GMAP (v.2021-08-25) was used to map the IsoSeq sequences (see the Supplementary Note for the parameter and filtering settings).

### Repetitive sequences annotation

De novo identification of carrot repetitive DNA was carried out with RepeatModeler (v.2.0.1) (http://www.repeatmasker.org/RepeatModeler/). The annotation of the consensus sequences was performed using a curated database of carrot LTR retrotransposons, Helitrons and MITE^61^, carrot satellite repeats^15^ and dicot plant repeats from RepBase (v.23.05)^62^ and DANTE (v.1.1.0)^63–65^. Masking was performed using RepeatMasker (v.4.1.0; http://www.repeatmasker.org) (see the Supplementary Note for the parameter and filtering settings). Identification, annotation and age analysis of LTR retrotransposons was performed as described by Kwolek et al.^66^ (see the Supplementary Note for the parameter and filtering settings). The quality of the assembled repetitive sequences was evaluated using the LTR Assembly Index, as recommended for comparison between assemblies of the same species^67^. For comparative analysis, all the repetitive sequence analyses were also performed using the DH1 v.2 genome assembly using the same methods outlined above. Carrot centromeric and telomeric repeats^15,68^ were mapped to the DH1 v.3 assembly using Blastn with the default parameters and dust set to ‘no’.

### Gene prediction and genome annotation

A multi-step approach was used to predict the most comprehensive gene model catalogue for the carrot genome v.3. MAKER (v.3.01.03)^69^ and GeMoMa (v.1.6)^70^ were used to perform gene prediction based on the integration of de novo gene prediction and evidence-based predictions. For MAKER, carrot expressed sequence tags^59^, DH1 Illumina and IsoSeq transcriptome sequences, gene models obtained from five closely related or model species (Supplementary Table 12), and proteins from Uniprot-sprot were used as transcript evidence. AUGUSTUS (v.2.5.5)^71^ and SNAP (commit of 3 June 2019)^72^ were used for de novo prediction (see the Supplementary Note for the details). Through this analysis, MAKER predicted 28,721 gene models. Next, GeMoMa was used to improve the quality of the splice junction sites predicted by MAKER and to predict the gene models that were not predicted by MAKER. The datasets included as input in GeMoMa were the predicted genes from the five related species or model species used for the MAKER prediction, the final gene models produced from the MAKER pipeline and splice sites mined from the mapping of the DH1 Illumina transcriptome data (see the Supplementary Note for the details) on DH1 v.3. This analysis produced an intermediate set of 32,625 gene models. A final step was performed to refine all gene models and predict any missing models. In this step, gene models predicted on the DH1 v.2 assembly^15^, named DCARv2 (32, 112) and RefSeq (44, 484), were transferred/re-predicted to the DH1 v.3 genome assembly using GMAP^73^ and GenomeThreader (v.2021-08-25)^74^. DCARv2 or RefSeq gene models that were not predicted by MAKER + GeMoMa, that had experimental evidence and that were not masked were considered as new gene models. In those cases where the structure of the RefSeq and DCARv2 gene models were not in agreement, the correct structure was manually inspected using the experimental evidence. Finally, high-quality IsoSeq transcripts were mapped to the DH1 v.3 assembly using GMAP and GenomeThreader. Those transcripts mapping with appropriate gene structure and not predicted in the previous steps were added to the gene model catalogue. In total, 3,586 gene models were added by manual curation and polishing, which resulted in a total of 36,211 gene models in the DH1 v.3 gene model catalogue (DCAR v.3.0 Gene Prediction) (Supplementary Tables 11 and 12).

Blast2Go^75^ was used to annotate the predicted gene models obtained from the last step using the NCBI, KEGG, InterPro and GO databases. PlantTFcat (downloaded in December 2020)^76^ and PRGdb (v.3.0)^77^ were used to predict the transcription factors and resistance genes in v.3 gene models, respectively, as well as the DCARv2 genes for comparison purposes. To assess the completeness of annotation, the predicted gene models were searched against the BUSCO (v.3)^78^ plant dataset (embryophyta_odb9) (Supplementary Table 13). An in silico search for the prediction of candidate microRNAs and small nuclear RNAs in the assembled genome was conducted by INFERNAL (v.1.1.2)^79^.

### Resequencing and phenotyping

For resequencing, a set of 542 cultivated carrots from the National Plant Germplasm System were grown from seed at the Hancock Agricultural Research Station (Hancock, WI, USA) during the summer of 2018 (Supplementary Table 22). An additional set of 88 wild carrots, chosen from the National Plant Germplasm System to represent multiple geographic origins, were grown from seed at the University of Wisconsin–Madison Walnut Street Greenhouse during the winter of 2018 (Supplementary Table 22). Roots were harvested with the tops attached, and mature leaf tissue was collected from each sample. Genomic DNA of each sample was extracted from lyophilized leaf tissue using the Machery-Nagel NucleoSpin Plant II Core kit. Paired-end libraries were sequenced on a NovaSeq6000 sequencer (Illumina) at the University of California, Davis, Genome Center in Davis, California.

Phenotyping for the resequencing material was performed on the basis of visual appearance and high-performance liquid chromatography (HPLC). At harvest, the presence of extensive lateral roots, root pigmentation and evidence of bolting were recorded and used as indicators to confirm the classification of accessions as wild. Visual colour scoring was completed for 630 carrot accessions by taking a cross-section of the taproot and assigning categorical scores of white, yellow, orange, red and purple (Supplementary Table 24). The concentrations of α-carotene, β-carotene, lutein and lycopene were quantitatively measured via HPLC in 528 accessions within three weeks of harvest. Within two weeks of harvest, slices were taken at mid-root, lyophilized and processed as in refs. ^80,81^ (see the Supplementary Note for the details). The HPLC data were filtered to remove samples with inconsistencies between technical replicates. Other samples were removed from downstream analyses if the HPLC data were not representative of the visual score. Carotenoid concentrations were reported in μg per g dry weight of tissue. This resulted in a set of 435 accessions with HPLC scores that were used for GWA analyses. Considering that the focus of this study was orange carotenoids and that α-carotene and β-carotene represent the major carotenoids in orange carrot, the ratio of α-carotene and β-carotene concentration was calculated relative to the total carotenoid concentration on a per-sample basis (Supplementary Fig. 8, Supplementary Table 27 and Supplementary Note). This method ensured that data across HPLC runs were normalized. The classification of Early and Improved cultivars in the different carrot root types was based on the description of the typical carrot shapes in ref. ^82^.

### Variant calls

Illumina reads from the 630 resequenced carrot accessions were mapped to the assembled genome with BWA (v.0.7.17–r1188) using the BWA-MEM algorithm. These alignments were used for variant calling following the Genome Analysis Toolkit (GATK, v.4.0.7.0) best practices^83^. Low-quality variants were removed using the following filters: minDP > 5, MQ < 40, FS > 60, QD < 2, MQRankSum < −12.5 and ReadPosRankSum < −8.0. Indels and non-biallelic sites were removed, and sample genotypes were filtered for a minimum GQ > 20. Finally, BCFtools (v.1.9)^84^ was used to remove singletons and sites with more than 20% missing data, leaving 23,375,112 SNPs across 630 samples. Removing variants with a minor allele frequency (MAF) <0.05 retained 5,393,228 SNPs across 630 samples, indicating that the majority of variants occur at a low frequency. For accurate estimates of nucleotide diversity, an allsites VCF that included invariant sites was also generated, with the same filtering criteria applied to SNPs and by removing low-quality invariant sites on the basis of the following filters: minDP > 5, QUAL < 30, MQ < 40, MQRankSum < −12.5 and ReadPosRankSum < −8.0.

### Population structure, phylogenetic analysis and PCA

To infer population ancestry, 300,981 SNPs were randomly sampled and LD pruned with a window size of 50 kb, a step size of five variants and a variance inflation factor of 2 using the command indep 50 5 2 in PLINK (v.1.90b3.44)^85^, resulting in 168,410 LD-pruned SNPs. Population structure was characterized using ADMIXTURE (v.1.3.0)^86,87^ on this LD-pruned SNP set. ADMIXTURE was run for *K* = 1 through *K* = 10 with a random number seed generated from the current time using the command admixture -s time. The coefficient of variation values for *K* = 1 through *K* = 10 were compared, and the *K* with the lowest coefficient of variation was chosen as the most optimal fit. Using this approach, the strongest support was identified for *K* = 5, but results at *K* = 6 were also explored (Supplementary Fig. 1).

Population genetic analyses were performed on a core set of 353 low-admixture samples, defined here as an ancestry coefficient >0.9 for a given reference population: wild (*n* = 52), Landrace-A (*n* = 30), Landrace-B (*n* = 73), Early cultivar (*n* = 42) and Improved cultivar (*n* = 156) (Supplementary Table 24). The phylogenetic analysis was performed on both the full set of all 630 samples and on the low-admixture set for comparison. For the low-admixture set, a neighbour-joining phylogeny was constructed with 110,780 LD-pruned SNPs using PHYLIP (v.3.696)^88^. A consensus of 1,000 bootstrap replicates was used to construct the resulting phylogeny. *D. syrticus* was used as an outgroup^89^. The resulting consensus tree was fitted over the original tree using a Perl script^90^. The phylogeny was visualized using the R package ggtree^91^. The same methodology was used for the full set of 630 samples, except for 10,000 LD-pruned SNPs and 100 replicates being used to construct the phylogeny.

PCA was performed using the function snpgdsPCA implemented in the R package SNPRelate (v.1.20.1)^92^ on the LD-pruned set of 168,410 SNPs with all 630 samples and for the set of 353 low-admixture samples.

### Gene flow, *f*_3_-statistic and *f*_4_-statistic analysis

Gene flow between populations was inferred by running TreeMix (v.1.12)^93^ on 26,670 LD-pruned SNPs with no missing data for the 353 low-admixture samples. The model was run with 100 replicates, each with 1,000 bootstraps for one to five migration edges. The most optimal number of migration edges was identified using OptM (v.0.1.6)^25^. Additionally, gene flow was assessed using *f*_4_-statistics by running the qpDstat program in AdmixTools v.7.0.2 (ref. ^94^). Population comparisons were set up as *f*_4_(outgroup, population X; population Y, population Z), where the outgroup included samples of *D. sahariensis* and *D. syrticus* and is not expected to have admixture with the test populations. Gene flow between test populations was considered significant if *Z*-scores had absolute values >3, with high negative values suggesting gene flow between test populations X and Y and high positive values suggesting gene flow between test populations X and Z.

To further clarify the relationships and relative divergence times among carrot subpopulations, outgroup *f*_3_-statistics were used to estimate the amount of shared genetic drift between pairs of populations relative to a distant outgroup comprising wild samples from related *D. carota* subspecies, which are genetically equidistant to the pair of populations being compared. The qp3Pop program in AdmixTools v.7.0.2 (ref. ^94^) was used to compute outgroup *f*_3_-statistics using the structure *f*_3_(reference population, test population; outgroup) with the option inbreed set to ‘YES’. Higher *f*_3_ values indicated a higher degree of genetic similarity and a longer shared branch length between the reference and test populations relative to the outgroup.

### Genetic diversity, *F*_ST_ and LD analysis

Pairwise *F*_ST_ and *π* were calculated within 100-kb windows using Pixy (v.1.2.7.beta1)^95^ and an allsites (variant and invariant sites) VCF as the input file (see https://github.com/dsenalik/Carrot_Genome_DH1_v3 for the details and parameters). Pairwise values were calculated for comparison of domesticated, improved and wild populations using the low-admixture set.

LD decay was calculated using 5,393,228 SNPs filtered for MAF < 0.05 among samples identified to have low-admixture proportions from each of the five populations. LD decay was calculated for all SNPs within 1-Mb windows using the command OutStat implemented in PopLDdecay (v.3.31)^96^.

### Demographic analysis

Estimates of effective population size history and divergence times were obtained using SMC++ software (v.1.15.2)^26^ (https://github.com/popgenmethods/smcpp), which uses a coalescent hidden Markov model to leverage information on LD and the site frequency spectrum from unphased genomic data. To reduce confounding due to gene flow, samples used in this analysis were restricted to the individuals with low admixture. The full set of 23,375,112 quality-filtered SNPs was included for demographic analysis to avoid excluding low-frequency sites and was filtered to exclude sites with ≥10% missing genotype calls using the command “view -e ‘F_MISSING > = 0.1’ -Oz” in bcftools (v.1.10.2)^97^. The resulting VCF file was converted to SMC format using the vcf2smc command in SMC++ and by treating repetitive sites identified by RepeatMasker (v.3.2.9) as missing data. To estimate a composite likelihood for population size histories and divergence times, distinct datasets were generated for each population by conditioning allele order across five randomly selected distinguished individuals. Population size history was estimated using the estimate command with the default parameters and a per-base-pair-per-generation mutation rate of *µ* = 4 × 10^−8^ as reported for *Lactuca sativa*^98^, which was the closest related species with a reported estimate for mutation rate. Divergence times were estimated by first generating a joint site frequency spectrum for each population pair using the vcf2smc command, followed by the split command. Estimate uncertainty for population size trajectories and divergence times was determined using a bootstrap approach in which ten replicates of the input genomic data for each distinguished individual were resampled in 5-Mb blocks. The code for the estimation of effective population size and divergence times using SMC++ (v.1.15.2) is available at https://github.com/mishaploid/carrot-demography.

### Genome-wide scans for signatures of selection

To identify regions of the genome that have undergone selection during domestication and improvement, we compared *F*_ST_, the ratio of nucleotide diversity and selective sweeps among pairwise comparisons of wild, domesticated and improved populations. Pixy software (v.1.2.7beta)^95^ was used to calculate *F*_ST_ and *π* across 100-kb windows for the low-admixture samples. An allsites VCF was used as the input to adequately distinguish between uncallable and invariant sites. XP-CLR (v.1.0)^99^ was then applied to identify variants that increased in frequency at a rate that is higher than by chance alone. XP-CLR scores were calculated using a set of one million variants filtered for MAF < 0.05, among all samples within the low-admixture dataset (Supplementary Table 24). XP-CLR scores were computed in a 0.05 cM window with a maximum of 100 SNPs per window and a 1-kb sliding window. If two SNPs were found to be highly correlated (>0.9), then their contribution to XP-CLR was downweighted. The top 2% of nucleotide diversity ratios between each of the five populations, the top 2% of *F*_ST_ values identified between each population and the genomic windows harbouring the top 1% of XP-CLR SNPs were merged, and regions that overlapped between all three analyses were identified as selective sweeps.

### GWA analysis

The phenotypic data for GWA analyses included HPLC data for the fraction of α + β-carotene and lutein to total carotenoids in addition to visual colour scores. The genotypic data were prepared and GWA analyses were completed on the US Department of Agriculture SCINet High Performance server. The genotypic data were filtered with vcftools (v.0.1.16)^100^ for sequencing depth of >5, MAF > 0.05, missing data <0.3, removal of indels, allele >2, and heterozygosity >0.3 and <0.7. Missing data from the genotypic file were imputed with Beagle (v.5.0) with the default settings^101^. The genotypic file was formatted in hapmap format with Tassel (v.5)^102^.

GWA analysis was completed using the R package GAPIT (2020.10.24 Gapit) v.3.0 (ref. ^103^) with multiple models tested, including a mixed linear model, multiple mixed linear models, and Bayesian-information and Linkage-disequilibrium Iteratively Nested Keyway^104^. The mixed linear model provided the best fit. Due to the number of SNPs used in the analyses, a random subset of 125,000 markers was used to complete a PCA and kinship analysis to account for population structure and relatedness, respectively. GAPIT code was also updated for computational speed by only writing results for the 100,000 markers most associated with each trait. The significance threshold was calculated using a modified Bonferroni correction in the R package simpleM^105^ for *P* < 0.05. Manhattan plots for the GWA results were generated using the R package qqman (v.0.1.8)^106^.

### RNA-seq analysis for *Or* and *Y2*

RNA-seq analysis was used to investigate the transcriptome profile of candidate genes underlying the *Or* and *Y2* loci mapped by GWA analysis. For the *Or* locus, RNA was extracted from three biological replicates of eight genotypes that were selected from a mapping population segregating for *Or*^17^(see the Supplementary Note for the details). Four genotypes represented plants that were homozygous for the orange cultivated allele (*Or_A*), and four represented plants that were homozygous for the wild allele (*Or_B*). Sequencing libraries were prepared using a TruSeq Stranded mRNA kit (Illumina), and the libraries were sequenced on a NovaSeq 6000 sequencer at the University of Wisconsin Biotechnology Center in Madison, Wisconsin. Transcriptome sequencing generated 1,091,729,253 reads across 24 samples (Supplementary Table 30).

For the *Y2* locus, existing RNA-seq data available in NCBI (BioProject PRJNA350691) were used in this study^16^. Transcriptome data represent six genotypes selected from a mapping population segregating for *Y2* (ref. ^16^). Three yellow genotypes were homozygous for the *Y2* wild allele, and three orange genotypes were homozygous for the *Y2* cultivated allele.

RNA-seq reads were first cleaned for adapter sequences using TRIMMOMATIC (v.0.36)^107^ and were then aligned to the reference genome using the package Rsubread (v.2.14.1)^108^. FeatureCounts (v.2.14.1)^109^ was then used to compute count matrices for each sample, and the results were then analysed using Limma (v.3.56.1)^110^. The analyses were performed in R v.3.5.0 (R Core Team, 2013). A log-fold-change testing threshold of 1.1 was used to identify genes with a substantial difference in observed log_2_-fold-change. The transcriptional interactome network analysis was performed as described in the Supplementary Note.

### Genetic effect and interaction analysis

Alternative genetic effects including additive effects, dominance, recessiveness and over-dominance for the ratio of α-carotene and β-carotene to total carotenoids were evaluated at a biallelic SNP locus (with reference and alternative alleles—for example, G and T) using SNPs with the maximum effect at the candidate genes *REC1*, *Or* and *EX1*, identified at QTLs mapped on chromosomes 2, 3 and 7, respectively. The SNPs and their locations in the DH1 v.3 genome used for this analysis were the following: A/C at position ch2_28364045, T/C at position chr3_5070341 and A/T at position chr7_39186121. To test for the additive and non-additive (dominance, recessiveness and over-dominance) effects, the SNPs were coded as 0 for homozygous reference allele (for example, AA), 1 for heterozygous (for example, AC) and 2 for the homozygous alternative allele (for example, CC). The allelic models were described by ref. ^111^.

All possible allele combinations were constructed for testing their interaction effect on the ratio of α-carotene and β-carotene to total carotenoids. To perform the analysis, the minimum number of alleles for each possible combination was set to five. All these analysis were performed in R using the lm function^112^.

### Reporting summary

Further information on research design is available in the Nature Portfolio Reporting Summary linked to this article.

### Supplementary information

Supplementary Information Supplementary Figs 1–10 and Note.

Reporting Summary

Supplementary Data 1 Supplementary Tables 1–46.

## Data Availability

The DH1 v.3 genome is available at CarrotOmics.org (ref. ^113^). All sequence data generated for this study were deposited in NCBI, under the umbrella BioProject PRJNA285926. The component BioProjects consist of PRJNA798760 for the reads used in the genome assembly, PRJNA865166 for the RNA-seq BioSamples and reads, and PRJNA865653 for the resequenced BioSamples and reads. The assembled genome sequences are available as accession numbers CP093343 through CP093353. The previously published reads used in this study are also available from the umbrella BioProject. Specific BioProject, BioSample and SRA accessions are also listed in the Supplementary Tables, where additional details for each dataset are provided. The Lunar White nucleotide sequences were deposited in NCBI under the name BankIt2620219 lunar_white_DCAR_730022_region, accession no. OP407851.

## Code availability

The list of the software and parameters used in this study are available through GitHub (https://github.com/dsenalik/Carrot_Genome_DH1_v3).
