# Supplementary material for: Population genomics identifies genetic signatures of carrot domestication and improvement and uncovers the origin of high-carotenoid orange carrots
Source: Nat Plants. 2023 Sep 28;9(10):1643–58. doi: 10.1038/s41477-023-01526-6 (PMC10581907; doi:10.1038/s41477-023-01526-6)
Supplement: Supplementary file 1 — Supplementary Figs 1–10 and Note. [file 41477_2023_1526_MOESM1_ESM.pdf]

nature plants

Article

https://doi.org/10.1038/s41477-023-01526-6

Population genomics identifies genetic signatures of carrot domestication and improvement and uncovers the origin of high-carotenoid orange carrots

In the format provided by the
authors and unedited

Table of Contents

1. Sequencing 4
   1.1. Long reads whole genome sequencing 4
   1.2. Paired reads Hi-C sequencing 5
   1.3. RNA sequencing 6
2. Genome assembly 6
   2.1. Phase I *De novo* assembly, scaffolding and polishing 7
   2.2. Phase II Pseudomolecule construction 8
   2.3. Phase III 10
3. Organelle genome assembly and annotation 11
   3.1. Identification of the organelle genome in the assembled sequences 11
   3.2. Annotation of the organelle genome 12
4. Assembly quality verification 12
   4.1. Analysis of GC content and contamination 12
   4.2. Evaluation of sequence correctness using PE data 13
   4.3. Evaluation of pseudomolecules and genetic linkage map collinearity 13
   4.4. Gene space coverage 14
5. Genome characterization and annotation 15
   5.1. Repetitive sequences 15
   5.2. Gene model prediction and annotation 16
   5.3. Isoform analysis 20
6. Testing Landrace_A-W as possible feral lineage 21
7. Selective sweep Enrichment analysis 22
8. Phenotyping plant material 22
9. RNA-sequencing analysis 23
10. Genotyping the Y2 insertion 24

References: 25

Supplementary Figures 30

Supplementary Figure 1. Model-based Population Structure of 630 resequenced carrot accessions. A) Barplot of admixture proportions at K = 5 and K=6. B) Accession's phenotypes representative of each population at K5 30

Supplementary Figure 2. TreeMix output for 2 migration edges. Migration edges are shaded based on the migration weight. 31

Supplementary Figure 3. Demographic history inferred by including the landrace A-wild accessions. 32

Supplementary Figure 4. PCA of Low Admixture Samples 33

Supplementary Figure 5. Neighbor joining phylogenetic tree of 630 samples. The consensus tree was constructed with 100 boostrap replicates. Branch tip colors represent the root color phenotypes and the outer bar corresponds to the population identity of each sample. The tree was rooted using *D. syrticus* as the outgroup. 34

Supplementary Figure 6. Neighbor joining phylogenetic tree of the low admixture set. The consensus tree was constructed with 1,000 boostrap replicates. Branch tip colors represent the root color phenotypes and the outer bar corresponds to the population identity of each sample.

The tree was rooted using *D. syrticus* as the outgroup. Bootstrap value >70% support are illustrated.. 35
Continue Supplementary Figure 6. Neighbor joining phylogenetic tree of the low admixture set. The consensus tree was constructed with 1,000 boostrap replicates. Branch tip colors represent the root color phenotypes and the outer bar corresponds to the population identity of each sample. The tree was rooted using *D. syrticus* as the outgroup. Bootstrap value >70% support are illustrated.. 36
Supplementary Figure 7. Values for population comparisons using f3-outgroup statistics as a measure of shared genetic drift between pairs of populations relative to a distantly related outgroup. Tests were represented as f3(reference population, test population; outgroup). The outgroup population contained wild samples from *Daucus carota* subspecies (subsp. *gummifer, maximus carota, and maritimus carota*), which are genetically equidistant to the test and reference populations. Higher f3 values indicate a higher degree of genetic similarity and a longer shared branch length between the reference and test populations relative to the outgroup. 37
Supplementary Figure 8. Interactome and enrichment analysis for gene DCAR_730022. a) Predicted Transcriptional interactome network representing significant interaction of DCAR_730022 with gene corresponding to photosystem II including response to singlet oxygen (DCAR_730007, DCAR_934511, DCAR_312895, DCAR_101854, DCAR_624272) and isoprenoid biosynthesis pathways (DCAR_933315, DCAR_624054, DCAR_415732, DCAR_522170, DCAR_519689, DCAR_832686, DCAR_418372, DCAR_623215) analyzed and visualized using Cytoscape. b) Heatmap representing expression-based enrichment (based on z-score value) of key genes having significant interactions with DCAR_730022 in the predicted. The significant interactions were observed between the DCAR_730022 with the genes involved in both Isoprenoid biosynthesis and photosystem related genes. In particular the gene has a significant interaction with DCAR_933315 (Undecaprenyl pyrophosphate synthase (UPPs) involved in the synthesis of Farnesyl Pyrophosphate (FPP) synthesis) and genes involved in GGP synthesis (DCAR_519689 & DCAR_832686) which act as precursor during carotenoid biosynthesis. 38
Supplementary Figure 9. Relationship between total carotenoid content and % of α- + β-carotene. A) Correlation between the ratio of α- + β-carotene concentration to total carotenoid concentration, or ABCR (ordinate) and the total carotenoid concentration (abscissa) in the carrot accessions evaluated in this study. Red and purple samples (n=29) were not included in this analysis. Based on Spearman's correlation, carotenoid concentration is strongly correlated with the ratio of α and β-carotene (two-sided, $p = 1.277345e-53$). B) The ABCR found in carrots visually phenotyped as dark orange (*n*=279), pale orange (*n*=38), white (*n*=20), and yellow (n=69), categories. Dark orange carrots had a significantly higher ratio of α and β-carotene (LSD, p-value < 0.001) than the other categories. Pale orange carrots also had a significantly higher (LSD, < p-value < 0.001) ratio of α- + β-carotene, relative to yellow and white carrots. Most yellow and all white carrots had a small ratio α and β-carotene and were not statistically different from each other. The boxplot represents the 25th, 50th and 75th percentiles with the upper and lower whisker 1.5x the 75th and 25th percentiles, respectively. 39
Supplementary Figure 10. Examples of pale orange (pOr) and dark Orange (dOr) phenotype harboring different allele combinations at the *Or* and *EX1* gene. pOr sample harbor a combination of wild and cultivated alleles at the two genes (e.g. A_B_) and dOr genotype harbored cultivated (A_A_) alleles at both genes. The graph illustrated in the up left corner of

each picture represent the percentage of lutein and α- +β-carotene relative to the total amount of carotenoids. 40
References 40

Supplementary Note

1. Sequencing

1.1. Long reads whole genome sequencing

Plant material: A doubled haploid orange Nantes type carrot (DH1, NCBI Biosample SAMN03216637), was used for whole genome sequencing with Pacific Biosciences (PacBio), Oxford Nanopore and Hi-C sequencing technologies. Carrot DH1 plants were grown in the greenhouse at the North Carolina Research Campus, Kannapolis, NC. For DNA extraction fresh unexpanded leaves were harvested, frozen in liquid nitrogen and stored at -80 ℃.

PacBio SMRT cells genomic DNA library preparation: High molecular weight DNA was extracted using the CTAB protocol as described by [1]. DNA quality and concentration were evaluated using gel electrophoresis and the Qubit dsDNA BR Assay Kit (Thermo Fisher Q32850). About 10μg of DNA was assessed using the Agilent TapeStation to assure the size of the DNA fragments were ≥60 kb. One SMRTbell library was generated using the SMRTbell Template Prep Kit (Cat 100-259-100) following the PacBio's ">20kb Template Preparation instruction and using BluePippin Size-Selection System (15-20kb) for Sequel Systems" procedure and checklist.

PacBio whole genome sequencing: Sequencing was performed using a Pacific Biosciences Sequel machine at the Genomics Laboratory at the DHMRI, Kannapolis, NC. In total eight SMRT cells were run yielding over 34 Gb of long read DNA sequences (~73× genome coverage) with an average subread length of >8.6kb (**table S1**). All the sequences from the eight SMRT cells were combined and used for the downstream analysis.

Oxford Nanopore genomic DNA library preparation: DNA was extracted using the CTAB method as described in [1] with some modifications to clean the DNA samples. Modifications included an RNAse treatment at 37 °C for 60 minutes to remove RNA, removing the NaOAc treatment to avoid salt contamination, adding an overnight step at -20 ℃ prior to final precipitation and washing the DNA with 70% ethanol three times. DNA quality and concentration were evaluated as described above. The libraries for Oxford Nanopore sequencing were prepared according to the library preparation protocol 1D Lambda Control Experiment (SQK-LSK1108).

Oxford Nanopore whole genome sequencing*:* Using the Oxford Nanopore real-time long read sequencing platform (FLO-MIN107 and SQK-LSK108) over 717 Mb sequences (~1.5× genome coverage) were generated, with an average subreads length of 5.3 Kb (Supplementary Table 1). Sequencing was performed at Plants for Human Health Institute (PHHI), Kannapolis, NC. The base calling was performed using the base caller script implemented in "ont-albacore" software package v2.3.1 [2].

1.2. Paired reads Hi-C sequencing

Plant material and library preparation*:* The Hi-C library for DH1 was prepared using the PhaseGenomics (Seattle, WA) Proximo Hi-C Plant Kit. Approximately 0.2g of young leaf tissues were collected from the DH1 plants, rinsed thoroughly in dH_2O, patted dry, and chopped finely in a petri dish using a scalpel. Petiole tissue was discarded. The finely chopped leaf tissue was used to create a Hi-C library according to the Proximo Hi-C Plant Kit protocol. The library concentration was determined using the Qubit 3.0 fluorometer (Life Technologies, Carlsbad, CA). QC was performed by Phase Genomics, using qPCR analysis and running the Agilent TapeStation (Agilent, Santa Clara, CA). An SPRI bead size-selection was performed using the right-hand selection protocol outlined in the SPRI select User Guide (Beckman Coulter, Brea, CA). After size-selection, the DH1 carrot library had an average fragment size of ~400 bp. The library had a molar concentration of 8.64 nM after size-selection and correcting for fragment size according to the qPCR run.

Hi-C sequencing*:* Hi-C libraries were sequenced by Novogene (Sacramento, CA) on one lane of the Illumina HiSeq 2500 using PE150 chemistry yielding to over 465M pair-end reads (~295× genome coverage). Raw reads were processed by trimming adaptor and low-quality sequences using Trimmomatic [3] considering the HiSeq adapters the following parameters: 2:30:10 LEADING:3 TRAILING:3 SLIDINGWINDOW:4:15 MINLEN:80. The trimmed and high quality sequences retained after this step were used for the downstream analysis (Supplementary Table 1).

In addition to the newly generated long reads (PacBio and Nanopore) and Hi-C reads, previously published [1] DH1 DNA sequences including 10 Kb, 20 Kb 40 Kb meta-pair reads, and a collection of BAC-end sequences (BES), were used for scaffolding and quality verification (Supplementary Table 2).

1.3. RNA sequencing

Plant material*:* For RNA extraction, fresh tissue from germinating seedlings, flower, phloem, xylem, petiole from 2 month old plants and leaf at two developmental stages (fully open and young unexpanded leaf), were collected in triplicates from DH1 carrot plants, frozen immediately in liquid nitrogen and stored at -80 °C.

PacBio IsoSeq total-RNA library preparation*:* Total RNA was extracted using the RNeasy Mini Kit (Qiagen, Valencia, CA, United States) in accordance with the manufacturer's protocol and treated with RNase-free DNase I (New England BioLabs, Ipswich, MA, United States). RNA integrity was analyzed using Agilent 2100 Bioanalyzer (Agilent, Santa Clara, CA, United States) and samples were pooled in equimolar amounts to generate four tissue specific SMRTbell® libraries corresponding to germinating seedlings (SD), phloem (PH), xylem (XY) and upper tissues (UP). The UP library included pooled (in equimolar amount) RNA from the leaf, petiole and flower samples. Each library was constructed according to the protocol for Iso-Seq™ Template Preparation for Sequel® Systems from PacBio (Pacific Biosciences, Menlo Park, CA, United States). In brief, for each library, 1 µg of total RNA was reverse transcribed using the SMARTer® PCR cDNA Synthesis Kit (Takara Bio USA, Inc.) and amplified with Prime STAR GXL DNA Polymerase (Takara Bio USA, Inc.). Amplicons were purified and size selected using 1× and 0.40× AMPure PB Beads and used to generate SMRTbell® libraries using the SMRTbellTM Template Prep Kit 1.0 (Pacific Biosciences, Menlo Park, CA, United States).

IsoSeq sequencing*:* IsoSeq sequencing was performed using a Pacific Biosciences Sequel machine at the Genomics Laboratory at the DHMRI, Kannapolis, NC. Each library was sequenced in two SMRT cells. In total 117 M reads were generated totaling 142 Gb of sequences (Supplementary Table 3**)**. All the sequences from the four libraries, and eight SMRT cells were processed and used for downstream analysis.

2. Genome assembly

A schematic view of the carrot genome assembly strategy is shown in Extended Data Fig. 1 and was summarized in three phases (I, II, II). The following terms are used to describe the carrot genome assembly DH1 v3.0. A contig is a contiguous genomic sequence that does not contain unknown bases (Ns) and that was not merged into scaffolds or superscaffolds. Scaftigs

are defined as all portions of a final assembly (superscaffolds, scaffolds, and contigs) consisting of contiguous sequence, with gapped sequences split into separate scaftigs at every occurrence of unknown bases (Ns). A scaffold is a portion of the genome sequence reconstructed from end-sequenced whole genome shotgun fragments and composed of contigs with associated gaps. The final assembly is non-redundant, i.e. sequences or portions of sequences used in higher level constructs are removed from the lower category, e.g. if a contig is present in a scaffold, it is no longer present in the final assembly as a contig. The nomenclature for sequences is DCARv3_Chr# for the nine chromosome pseudomolecules, in which DCAR indicates carrot genome, v3 represents the third version of the genome assembly, and "#' is the unique numeric identifier for the sequence. Scaffold, contig and scaftig terminology was used only to describe the statistics of the assembly and not to label sequences in the final assembly.

2.1. Phase I *De novo* assembly, scaffolding and polishing

***De novo genome assembly*: ** The *de novo* genome assembly of DH1 carrot was conducted using all raw PacBio long reads sequences obtained from the eight SMRT cells (See section 1.1 and Supplementary Table 1). FALCON (v.0.3.0) [4] and CANU (V.1.5) [5] assemblers were independently used for *de novo* genome assembly. The FALCON genome assembly was performed using the Bioinformatics Research Computing Cluster (BRC) at North Carolina State University using 17 nodes (each 32 cores and 64GB RAM) using the following parameters: length_cutoff=5000, length_cutoff_pr=1000, --max_diff=50, --max_cov=50, --min_cov=4. The CANU assembly was performed using a VRTX machine located at Plants for Human health Institute (PHHI), Kannapolis NC, with 72 cores and 200GB RAM using the default parameters.

The statistics of each assembly are reported in Supplementary Table 4. The results of the two assemblies indicated that CANU assembled a larger portion of the carrot genome, with a higher contig N50. Indeed the CANU assembly represented about 34 Mb (436 Mb vs 402 Mb) of extra sequence and had a 3.7 fold longer (3.36 Mb vs 0.89 Mb) contig N50 compared to the FALCON assembly. Also the CANU assembly covered the sequences assembled by the FALCON assembly.

***Genome sequence polishing*: ** Although the CANU assembler includes a step to correct the PacBio reads as a part of its pipeline, two extra rounds of polishing were performed to further improve the quality of the assembled sequences. The assembly contigs were polished by aligning

the raw PacBio reads to the assembly, and correcting the sequencing errors using the Arrow algorithm implemented in GenomicConsensus (https://github.com/PacificBiosciences/GenomicConsensus). To further improve the assembly, another round of polishing was performed by aligning the Hi-C short reads to the assembly and correcting the sequencing errors using Pilon (v1.23) [6] (Supplementary Table 4), using default parameters.

Scaffolding**:** The high quality Hi-C reads were aligned to the polished contigs using BWA mem (0.7.17) [7] and the mapping information was used to cluster and order the contigs into scaffolds using SALSA2 (v2.3) scaffolder [8]. SALSA2 was executed with 200 iterations and GATC as the restriction site. SALSA2 also broke the contigs into sub-contigs when a significant number of discordant mapping reads indicating chimeric sequences were detected (Supplementary Table 4).

A number of unknown nucleotides ("N"s) separated the sequence of the contigs assembled into a scaffold. The number of Ns were estimated by SALSA2 considering the paired-end read insert size in the genome assembly. Contigs that were not assembled into scaffolds in this step remained as single contigs (Singletons).

Gap filling I**:** To maximize the use long-read sequences, gaps represented by unknown nucleotides (Ns) were filled by PBjelly (v15.8.24) [9] using high quality PacBio and nanopore reads. The SALSA scaffolded assembly (see section 2.3) was used as input file and PBJelly was set with the following parameters: (--minMatch 8 --minPctIdentity 80 --bestn 1 --nCandidates 20 --maxScore -500 --nproc 39 --noSplitSubreads).

Overall, 131 gaps were closed and 414 contigs were extended, adding ~2.6 Mb of known sequences (~0.118 Mb replaced with Ns and ~2.5 Mb contigs extension) (Supplementary Table 45). This resulted in a significant improvement of the genome sequence contiguity (+ 1.9 Mb contig N50) (Table 1 and Supplementary Table 4).

2.2. Phase II Pseudomolecule construction

Anchoring and chimeric sequence correction**:** In this step, multiple data were integrated to identify and correct chimeric sequences, ordering and orienting all the sequences into their corresponding chromosome pseudo-molecules.

A high-quality genetic map (2,075 markers) and its bin map (918 bins each representing a true recombination event)[1] were used to anchor and orient the contigs and scaffolds into pseudomolecules. BWA mem [7] was used to map the marker sequences to the indexed genome produced in section 2.1 The sequence alignment/map (SAM) file was filtered for MAPQ>=10 and marker sequences with mapping frequency of >1 were discarded. The resulting high-quality and uniquely mapped markers were then integrated with their associated genetic linkage groups. In total, 1,993(96%) markers and 873(95%) bin markers were uniquely mapped.

This analysis was coupled with the information from 10 kb, 20 kb, and 40 kb meta-pair reads (MPE), a collection of BAC-end sequences (BES) and the Hi-C heatmap information (Supplementary Table 2). STAR aligner (v2.7.10a) [10] was used to map the meta-pair reads to the indexed genome produced in section 2.1. BWA mem [7] was used to map the BES to the indexed genome produced in section 2.1. All the SAM files were then filtered for MAPQ >=10 and the reads that had both pairs mapping were kept for the downstream analysis. The Hi-C reads were processed as follows:

- mapping the high-quality Hi-C reads pre-processed in section 1.2 on the genome assembly obtained from section 2.1 using BWA mem [7] considering the following parameters (-5 -t 44);
- extracting the mapped reads with MAPQ >= 10 and order of R1, chr1, pos1,0, R2, chr2, pos2, 1, mapq1, mapq2 in tab delimited format;
- Sorting the output using a custom command (sort -k3,3d -k7,7d);
- Running juicer_tools.1.8.9_jcuda.0.8.jar included in the Juicer package [11] considering pre –q 10 (.hic file);

IGV [12] was used to visualize the mapped MPE, BES and markers, and JuiceBox [13] was used to visualize the Hi-C heatmap. A custom program was used to visualize and identify connections of PE sequences (PE BACs, 10, 20 and 40 kb MPE) to other scaffolds or contigs. Construction of the pseudo-molecules was initiated using scaffolds containing sequences of mapped markers. Scaffold connections supported by MPE and BES were used to connect the sequences. During this process the quality of each scaffold assembly and contiguity was verified by visually inspecting the coverage of large insert libraries (20 and 40 kb) and the consistency of the Hi-C signals along the diagonal and of marker order along the linkage map.
Possible chimeric sequences were identified as:

- Scaffolds or contigs containing sequences of markers mapped to different LGs or to distal locations of the same LG;
- Scaffolds or contigs with regions not covered by MPE and BES sequences;
- Sequences with no interaction signals along the Hi-C heatmap diagonal;

Within each chimeric sequence, the chimeric region was identified as those sequences not covered by MPE or PE-BAC sequences and with connection to other distal locations. Those regions were than manually inspected. The mid-point between the closest unambiguously aligned MPE sequences flanking the chimeric region was defined as the misassembly point. The corrected sequences were then used to progressively construct pseudomolecules.

Using this approach, 27 misassembled/chimeric regions were corrected and 389 Mb (164 contigs) were anchored, ordered and oriented into the 9 carrot chromosomes. This resulted in a genome sequence assembly with a scaffold N50 of 44.02 Mb (Supplementary Table 4).

2.3. Phase III

***Guided assembly*:** RaGOO (v1.1) [14] was used to further improve the contiguity of the genome assembly and to anchor any additional non-anchored sequences. In this process, the contig-level version (scaftigs and contigs) of the genome obtained from section 2.1 were used with the scaffold-level version of the same genome (section 2.2) considering the following parameters (-C -t 43 -i 0.3). In this step, the order and orientation of all the anchored and oriented scaftigs were not altered, and the 409 non-anchored sequences (largely telomeric and centromeric sequences based on the RepeatMasker analysis) were anchored to the 9 pseudomolecules (Supplementary Table 5). Hence, a fully anchored and oriented genome assembly sequence with a scaffold N50 of 51.16 Mb was obtained.

***Gap Filling II*:** LR_Gapcloser (v3, 29 June 2007) [15], a tiling path-based gap closer that uses long reads to close gaps in a genome assembly, was used to fill the gaps across the pseudomolecules (scaffolds) considering the following parameters (-s p -t 40 -r 5). The assembly obtained from the previous step and the PacBio and Nanopore reads were used as input files for this analysis.

Overall, 138 gaps were closed and 211 contigs were extended, adding ~97kb of known sequences (~96kb replaced with Ns and ~1.5kb contigs extension) (Supplementary Table 45).

This resulted in a slight improvement of the genome sequence contiguity (+0.3 Mb contig N50) (Supplementary Table 4).

The final genome assembly v3.0 (hereafter DH1 v3) sequence covers >440 Mb, with a contig N50 >6 Mb, all assembled into 9 pseudomolecules/chromosomes (Supplementary Tables 4 and 5**)**. Compared with the previous assembly, DH1 v2.0 (hereafter DH1 v2), the v3 assembly includes about 12% (54 Mb) novel nucleotide sequences, >21% (>100 Mb) additionally anchored nucleotide sequences at the chromosome level, and represents a >193 fold increase in contig N50 (Table 1). The genome assembly contains only 554 gaps containing only 55.4 Kb of unknown sequence (Ns). The length of each chromosome ranged from 33.4 Mb (Chr. 8) to 64.5 Mb (Chr. 3)(Supplementary Table 5**).** The DH1 genome assembly v3 was used for all downstream analyses.

3. Organelle genome assembly and annotation

3.1. Identification of the organelle genome in the assembled sequences

The organelle genomes of carrot DH1 were identified and extracted from the polished CANU sequences and were kept and analyzed independently and not reported in Supplementary Table 4. The result of the analysis for the carrot organelle genomes are presented below.

To identify the chloroplast and mitochondrial sequences in DH1 v3 genome, DCARv2 plastid (NCBI accession number LNRQ01000011.1) and mitochondrial (NCBI accession number LNRQ01000010.1) sequences were tested against all the CANU assembled contigs. MUMmer 4 [16] was used to identify the matching sequences and generate the collinearity plots. Interestingly, only one sequence (contig 12) had a clear match versus both sequences with no overlapping region. In fact, CANU assembled the DH1 v3 plastid and mitochondrial sequences both side by side in one sequence (contig 12). This allowed us to clearly establish the DH1 v3.0 organellar genomes for the downstream analysis.

The two organellar genomes were separately extracted from this contig. The circular plastid sequence was rearranged to follow the usual convention of starting with the large single copy region. The mitochondrial sequence, also a circular molecule, was rearranged to match the convention of the DCARv2 assembly, with the mitochondrial plasmid region at the 3’ end. Sequences and overlaps of the circular molecule ends were verified by examining mapped reads.

Comparing the organelle genomes of v2 versus v3, the v2 plastid and mitochondrial had one and two gaps (unknown sequence regions), respectively, while these gaps were filled with known sequences in the DH1 v3 organelle genomes. One mitochondrial region, 91 nt in v2 (DCARv2_MT:142210..142300) and 162 nt in v2.0 (DH1 v3.0_MT:146735..146896), was examined and determined to be a tandem repeat that was missed in the v2 assembly, since this repeat is slightly larger than the read length used for the v2 assembly. In total, v2 plastid and mitochondrial sequences had assembled lengths of 155,848 and 244,980 nt, respectively while the DH1 v3 plastid and mitochondrial sequences have assembled lengths of 155,839 and 250,368 nt. In addition to other small corrections, this new assembly completed the mitochondrial circular genome by adding 9,913 nt of new sequence linking the ends of the incomplete v2 sequence. The sequence of the final DH1 plastid and mitochondrial genomes were deposited in NCBI (BioProject PRJNA798760; accession number, Plastid = CP093353; Mitochondrion = CP093352).

3.2. Annotation of the organelle genome

Gene annotations from the DCARv2 genome, sequences LNRQ01000010 (MT) and LNRQ01000011 (PT) were transferred to the v3 genome using UCSC Liftover [17]. Sequences of the gene predictions were compared, and were found identical between the assemblies, with the exception of one intron in DCAR_032448 (v2) vs. DCAR_M036238 (v3) rps3 containing a 17 nt insertion. New sequence in the DH1 v3 mitochondrial genome not present in the v2 assembly was evaluated for additional genes, but none were found.

4. Assembly quality verification

The reliability of reference sequence data is crucial for the interpretation of downstream structural and functional genomic analysis. Thus, a comprehensive analysis was carried out to evaluate the quality of the final carrot DH1 v3 genome assembly.

4.1. Analysis of GC content and contamination

To evaluate and verify the quality of the DH1 v3.0 genome assembly, GC content distribution and sequence contamination analyses were carried out.

Sequence contamination can bias the average GC content across the whole genome sequence. To evaluate the quality of the assembled sequence from this perspective, a binning approach to

examine the average GC content of every 10 kb non-overlapping window covering the whole genome sequence was carried out using a custom script. The analysis revealed the average GC content of 34.9% (STD ± 3.1) across the genome with no abnormal GC content value for any specific regions (Extended Data Fig. 5).

Fastq-Screen (v0.4.14) [18], a bioinformatics tool for detection of contamination in sequence data was used to examine the DH1 v3 genome sequence for any possible sequence contamination. Fastq-Screen included the databases of Human hg-38 (ftp.ensembl.org/pub/current/fasta/homo_sapiens/dna/), Mouse mm10 (ftp.ensembl.org/pub/current/fasta/mus_musculus/dna/), *E. coli* (sequence available from EMBL accession U00096.2), phiX (sequence available from Refseq accession NC_001422.1), NCBI UniVec (http://www.ncbi.nlm.nih.gov/VecScreen/UniVec.html), and the Illumina sequence adapters (sequence derived from the FastQC contaminants file found at: www.bioinformatics.babraham.ac.uk/projects/fastqc). No sequence contamination was observed in this analysis.

4.2. Evaluation of sequence correctness using PE data

Assuming that the distance between two ends of a paired-end sequence (PE) represents their true physical distance, we estimated the observed distance between the two end sequences of the BES-PE data based on their alignment against the carrot genome assembly. For this analysis we used 4,717 PE BACs that unambiguously aligned (filtering parameters described in 2.2) with both ends to the carrot genome assembly DH1 v3 and that were not used during the assembly process (in section 2.2). The fraction of PE data that aligned within the expected library insert size should reflect the fraction of assembled sequences that are consistently contiguous and correctly assembled. The results of this analysis were expressed as percent of PE reads that aligned within the average estimated insert size of the PE library, plus/minus twice the standard deviation. Overall, 9,213 (99.5%) PE BACs, unambiguously aligned within the estimated library insert size (Supplementary Table 6). In addition, the Hi-C heatmap showed a uniform distribution of genomic interactions along the diagonal, demonstrating the proximity of the assembled sequences and the quality of the assembly (Supplementary Fig. 2).

4.3. Evaluation of pseudomolecules and genetic linkage map collinearity

To independently verify the order of the scaftigs along the nine pseudomolecules (chromosomes), an independent high-quality genetic map (different from the one used for the anchoring and orienting process, section 2.2) was used. The linkage map was developed from population 3242 and included 989 non-redundant markers [19]. Marker sequences were mapped against the DH1 v3 assembly using BWA mem [7]. Reads with MAPQ<10 and/or multiple mapping were discarded and only high-quality uniquely mapped reads were retained for this analysis. The results revealed that the DH1 v3 assembly and the linkage map were highly collinear confirming the quality of the assembly (Extended Data Fig. 3).

4.4. Gene space coverage

The following transcriptome data and mapping strategy were used to assess gene space coverage of the carrot DH1 v3 genome assembly:

- a collection of 18,137 consensus carrot expressed sequence tags (ESTs) from [20] were aligned to the genome using BWA mem. Only ESTs with MAPQ>=10 were included;
- a collection of 512.9M RNA-Seq reads from 20 libraries representing expressed sequences from 20 different DH1 carrot tissues, developmental conditions or developmental stages (NCBI BioSamples SAMN03965304 – SAMN03965323)[1] were mapped and assembled with StringTie (v1.3.5) [21];
- a collection of 248,122 high-quality IsoSeq full-length transcripts were mapped to the carrot genome assembly using GMAP (v2021-08-25) [22] with the option –f 2 activating the splice aware prediction and mapping.

These analyses indicated that ~99.04% of the Illumina reads, 96.7% of the ESTs and 99.95% of the high-quality full-length IsoSeq transcripts (Supplementary Tables 7 and 8) aligned to the carrot genome assembly, providing evidence that the assembly covers the majority of gene space.

Together, the statistics and verifications provided strong evidence that the DH1 v3 genome assembly is of high quality. Compared to the DH1 v2 genome, DH1 v3 has higher quality in terms of genome coverage, sequence contiguity length and sequence completeness (Table 1).

5. Genome characterization and annotation

5.1. Repetitive sequences

To compare the repetitive content of the v2 and v3 genomes, a new annotation of the repetitive sequences in the two genomes was performed using the same method described here.

De novo identification of carrot repetitive DNA was carried out with RepeatModeler (v.2.0.1) (http://www.repeatmasker.org/RepeatModeler/). The consensus sequences obtained from RepeatModeler were aligned against a curated databases of carrot LTR-RTs, Helitrons, a previously published carrot MITE database [23], carrot satellite repeats reported by [1], and dicot plant repeats from RepBase (v.23.05) [24]. Redundant elements (80% identity across minimum 80% length) were removed from the final carrot repetitive DNA database. The remaining consensus sequences were annotated based on the presence of specific domains typical for particular groups of transposable elements using DANTE, a tool implemented in the RepeatExplorer pipeline [25-27]. Next, the carrot genome was masked with RepeatMasker (v.4.1.0; http://www.repeatmasker.org) using the non-redundant carrot repetitive DNA library.

Identification, annotation, and age analysis of LTR-RTs was performed as described by [28] In brief, LTR-RTs were mined using LTRharvest (GenomeTools v.1.6.1) [29] with parameters: -seed 80 -maxlenltr 4000 -mindistltr 3000 -mintsd 2 -maxtsd 20 -motif tgca. Clustering of LTR sequences (--ident 0.6 --overlap 0.7), used for determination of subfamilies was performed as described by SiLiX (v1.2.9) [30]. Annotation of LTR-RTs was performed by identification of conserved domains using DANTE (v.1.1.0) [25-27]. The age of LTR-RTs was evaluated based on the DNA distance of LTRs calculated from alignment created with Mafft (v.7.471) [31] in R 'ape' (v.5.4-1) package [32], using the "K80" model with default parameters. Ages were calculated using the formula T=K/2r, with the substitution rate of 1.3×10–8 per site per year, as proposed by [33] and [34]. Helitrons were mined using HelitronScaner [35] with default settings, and annotated using DANTE [25-27].

Repetitive sequences accounted for 49.5% (234 Mb) of the estimated genome size, including 53.1 Mb (11.3%) more repetitive sequences than predicted in the DH1 v2 genome assembly (Table 1 and Supplementary Table 9). The majority of the newly annotated repetitive sequences were long terminal repeat (LTR) elements, mainly located in centromeric and pericentromeric

regions (Fig. 1a). A total of 8,063 full-length LTRs were predicted in DH1 v3, 25% more than in DH1 v2. The increase in the number and cumulative length of full-length LTRs was particularly evident for the most abundant lineages, DcSIRE (Ty1/Copia superfamily) and DcRetand, DcAthila, DcTekay (Ty3/Gypsy superfamily) (Extended Data Fig. 4). The higher GC content (36-47%) of these LTR families, contributed to a higher GC content observed in newly assembled sequences (Extended Data Fig. 5, Supplementary Table 10). As expected, the most abundant newly assembled and annotated LTR lineages DcSIRE, DcRetand, DcAthila were also the younger elements, which likely represent genomic regions with high similarity, that were not fully assembled in the DH1 v2 assembly (Extended Data Fig. 6). The raw LTR Assembly Index (raw LAI), a standardized metric for comparison between assemblies 22 was much higher for DH1 v3 (22.88) as compared to v2 (5.09). These differences were consistent across all chromosomes (Extended Data Fig. 7), reflecting markedly improved contiguity of the current assembly.

5.2. Gene model prediction and annotation

5.2.1. Gene model prediction with MAKER

Gene prediction in the DH1 v3 genome was based on the integration of *de novo* gene prediction and evidence-based predictions. This process included several iterations of homology-based and *in silico* gene prediction steps, using short and long read sequences (Illumina and PacBio) as well as gene models obtained from multiple closely related species and model organisms.

The following transcriptome datasets were used as evidence in the gene model prediction:

- A collection of 512.9M RNA-Seq reads from 20 sequencing libraries representing expressed sequences from 20 different DH1 carrot tissues, developmental conditions or developmental stages (NCBI BioSamples SAMN03965304– SAMN03965323)[1]. These sequences were aligned against the DH1 v3 genome sequence using STAR (v2.7.10a) [10] aligner with the following parameters: --outSAMstrandField intronMotif --outSAMattrIHstart 0 --outFilterMismatchNmax 2 --outSAMtype BAM SortedByCoordinate. The resulting bam files were used as input for StringTie (v1.3.5) (https://ccb.jhu.edu/software/stringtie/) [21] to obtain Illumina base transcripts assembly,

using the following parameters: -m 50 -c 1 -f 0.01. The resulting GFF3 file was then used as an experimental evidence for the downstream analysis;

- A collection of 18,137 consensus carrot expressed sequence tags (ESTs) from [36];
- PacBio long-read IsoSeq transcript sequences developed in this study (**table S3**) were processed using IsoSeq3 (v3.3.0) processing pipeline and protocol developed by Pacific Biosciences available at https://github.com/PacificBiosciences/IsoSeq. The analysis resulted in a collection of 248,122 high-quality full-length transcript sequences per each library. High-quality full-length transcripts obtained in this step were mapped against the DH1 v3 genome assembly using GMAP (v2021-08-25) [22] considering the following parameters: –f= 2; and minimum identity and coverage= 99%. The GFF3 file obtained from this step was later used for gene loci identification, gene/isoform models prediction and correction of *in silico* predicted structures across the DH1 v3 genome assembly.

MAKER (v3.01.03) [37], was used to predict genes in the DH1 v3 genome. Initially, the repeats library constructed in section 5.1 was used as the input repeat database for MAKER pipeline, excluding the SIMPLE elements and allowing MAKER to soft mask them for better efficiency and gene model identification For MAKER parameters see GitHub page https://github.com/dsenalik/Carrot_Genome_DH1_v3. Next, MAKER was run in empirical mode to identify putative gene loci and structure using sequence similarity search and experimental evidences. Model training and *de novo* prediction of gene models was carried out using AUGUSTUS (v2.5.5) [38] and SNAP (Commit of June 3, 2019) (https://github.com/KorfLab/SNAP). Both programs were trained after each iteration using the following resources:

- High-quality and polished IsoSeq full-length transcripts from DH1 described above;
- High-quality and manually curated gene model prediction dataset from kiwifruit (*Actinidia chinensis* var. *chinensis*) genome obtained from [39];
- *Arabidopsis thaliana* gene models (Araport11 release, https://www.arabidopsis.org/download/index-auto.jsp?dir=/download_files/Genes/)
- Lettuce (*Lactuca sativa*) gene models (L. sativa v8, http://lgr.genomecenter.ucdavis.edu/Private/Downloads/Downloads.php),
- *Panax ginseng* gene models (IPGA v1.1, http://ginsengdb.snu.ac.kr/data.php),

- Tomato (*Solanum lycopersicum*) (ITAG3.2, ftp://ftp.solgenomics.net/genomes/Solanum_lycopersicum/annotation/ITAG3.2_release/)
- Protein sequences from the reviewed section of Uniprot-sprot (downloaded on December 1, 2019).

Carrot Illumina assembled transcripts were also used as a training sets, however preliminary results indicated that this data was introducing a large number of false gene models. Hence, these sequences were removed from the training set. MAKER was run with three iterations, and after each iteration the program was re-trained. After each iteration, predicted gene models were loaded into the IGV for quality verification and for tuning the parameters. MAKER based prediction produced 28,721 gene models. These gene models were then used to conduct the final homology based gene model prediction.

5.2.2. Gene model prediction with GeMoMa

GeMoMa (v1.6) [40], a homology-based gene prediction program that predicts gene models in target species based on gene models in evolutionary related reference species, was used to improve the quality of the splice junction sites predicted by MAKER and to predict the gene models that were not predicted by MAKER. GeMoMa was run using default parameters. The datasets included as input in GeMoMa were: predicted genes from related species described in section 5.3.1; final gene models produced from MAKER pipeline; Illumina splice sites to enhance the splice site prediction of the genes on the genome. This analysis produced an intermediate set of 32,625 gene models that integrated MAKER based prediction + GeMoMa base prediction. The set of gene models were used for final gene model curation.

5.2.3. Gene model curation

In addition to the *in silico* and homology base gene model predictions described above, a multi-step approach was used to generate the most comprehensive gene model catalog for the carrot genome DH1 v3. DCARv2 (32,112) and RefSeq (44,484) gene models predicted on DCARv2 were transferred to the v3 genome sequence. This step was performed using GMAP [22] and GenomeThreader (V2021-08-25) [41] that are two independent splice aware gene model predictors. The predicted loci were compared with the predicted v3 gene models explained before. DCARv2 or RefSeqgenes that were successfully transferred/predicted on the v3 but not

predicted by Marker and GeMoMa (see above) and that had at least one experimental evidence not masked by the repetitive sequences, were added in the v3 gene model catalog. In those case where the structure of the GMAP and GenomeThreader based predictions and/or/ the RefSeq and the DCARv2 gene models were not in agreement, the model was manually checked and the structure confirmed by the experimental evidence was considered as the final prediction. In addition, the full-length high-quality IsoSeq transcripts (248,122) were transferred to the v3 genome sequence (using GMAP and GenomeThreader as two independent splice aware gene model predictors) and the predicted loci was compared with the predicted v3 gene models explained before. Those IsoSeq full-length transcripts with appropriate gene structure that were not masked with repetitive sequences, and not yet predicted by all method described above, were also added to the catalog. In total, 3,591 gene models were added in this manual curation and polishing which resulted to the total of 36,211 gene models in the v3 gene model catalog (Supplementary Tables 11 and 12).

5.2.4. Gene annotation and quality verification

Blast2Go (OmicsBox 3.0.29) [42] was used to annotate the predicted gene models obtained from the last step using the NCBI, KEGG, InterPro, and GO databases. In total, 99.5%, 3.4%, 0.8%, 94.2%, and 91.5% genes were annotated using the NCBI nr [43], GO [44], KEGG Pathway [45], InterPro [46] and InterPro GO databases [47], respectively. This allowed the assignment of 34,297 genes with potential functions (Supplementary Table 14). The remaining 271 genes were labeled as putative proteins in the gene model prediction and annotation catalog.

PlantTFcat (Downloaded on Dec 2020) [48] a reference plant transcription factor and transcriptional regulator categorization tool, was used to predict the transcription factors and regulatory genes in v3 gene models as well as the DCARv2 genes for comparison purposes. The summary tables of the transcription factor genes annotated in v3 and v2 as well as their comparison presented in Supplementary Tables 20 and 21**.**

PRGdb (v3.0) [49], a comprehensive platform for prediction and analysis of plant disease resistance genes, was used to predict the resistant genes in v3 as well as the v2 for the comparison purposes. The summary table of the disease resistant annotated gene models from v3 and DCARv2 as well as the comparison are presented in Supplementary Tables 22 and 23**.**

To assess the completeness of annotation, the predicted gene models were searched against the BUSCO v.3 [50] plant dataset (embryophyta_odb9) (Supplementary Table 13).

5.2.5. Non-coding RNAs prediction and annotation

An *in silico* search for prediction of candidate microRNAs and snRNAs in the assembled genome was conducted by INFERNAL (v1.1.2) [51] software, implementing the cm search function against Rfam database (v13.0) and the p-value of <0.05. Among 49,638 putative ncRNAs candidates form INFERNAL table output, 9,963 high confident ncRNAs were obtained and the summary report is presented in Table 1.

5.2.6. GC content analysis

To explore the structural aspects of the novel sequences in the new assembly, we evaluated the GC content. At the whole-genome level, sequences with a guanine-cytosine (GC) content of ~34.0% were dominantly abundant in both v2 and v3 assemblies; however, the distribution of GC content among novel sequences peaked at ~35.5% (Extended Data Fig. 5a). The GC content of the gens predicted in the newly assembled sequences was lower than the GC content of the all genes predicted in the v3 genome (Extended Data Fig. 5b). These results indicated that non genic sequences likely repetitive sequences are contributing to the higher GC content observed within the newly assembled sequences. Indeed, as indicated above, newly assembled regions are enriched with TE families that have a very high GC content (Supplementary Table 10).

5.3. Isoform analysis

In order to verify the reliability of the IsoSeq data and isoform detected using Sqanti3 (v4.1) pipeline [52], eight gene models presenting two distinct isoforms were selected to quantify their level of expression using RT-qPCR. In brief, mRNA, from the same DH1 tissues used to generate the IsoSeq sequences was extracted using Trizol reagent (Invitrogen, Carlsbad, CA, United States) and treated with RNase-free DNase I (New England BioLabs, Ipswich, MA, United States). Then mRNA was reverse transcribed into cDNA and amplified using primer pairs specific to each isoform (Supplementary Table 16). The selected isoforms belong to the following four different alternative splicing categories detected by bioinformatics analysis were tested: intron retention (IR), exon skipping (ES), alternative 3'-end (A3End), and alternative 3'-splicing site (A3Site) (Extended Data Fig. 9). qRT-PCR and sequencing were performed as described by [53]. The Actin gene was used as an internal control to calculate the relative expression levels of each gene of interest by the 2-ΔCT method and multiplied by 1,000 (formula

1,000 * 2-ΔCT) to enhance readability [54,55]. Statistical analysis was performed using a one-way analysis of variance (ANOVA) using the software Statistical Product and Service Solutions (SPSS) v 23 (IBM, NY) followed by Tukey's HSD test.

The structures of all 8 loci's isoforms were confirmed (Extended Data Fig. 10). The relative expression level of each isoform at a specific locus correlates with the percentage of unique Circular Consensus Sequence (CCS) detected in the IsoSeq experiment. For instance, DcHpxo (DCAR_310121) is subject to an alternative splicing of its 4th exon which was only detected in about 10% of the transcripts by both IsoSeq and RTqPCR experiments (Extended Data Fig. 10). For two loci (GST- DCAR_124495 and TPS- DCAR_113298), the conserved protein domain is lost in one of the isoform which could have impact its function, highlighting a potential functional role of these isoforms. For instance, in mosquitos, a GST was found to be regulated through alternative splicing with five coding exons that are alternatively spliced to produce four different mature GST transcripts [56]. TPS are the key enzymes responsible for the biosynthesis of terpenes [57]. Our RT-qPCR, and IsoSeq data, revealed that TPS- DCAR_113298 is specifically expressed in the aerial tissues with a third of its transcripts producing potentially no functional proteins, an interesting candidate to further investigate the function of alternative splicing with a potential role in the aroma of carrot and closely related aromatic plants from the Apiaceae family.

6. Testing Landrace_A-W as possible feral lineage

The high level of admixture between Landrace-AW accessions and Landrace-A observed in the population structure analysis suggests that gene flow between wild and cultivated accessions occurred during the carrot improvement process. To test this hypothesis, SNPs from all populations (low admixture set) were analyzed using TreeMix (v1.12) [58]. The topology of the population graph matched the relationship established by the phylogeny (Supplementary Fig. 2). Gene flow from the Wild population to Landrace-AW was detected. In addition, the TreeMix results suggest the presence of gene flow from the Early cultivar group to the Wild population. Gene flow between these populations is also reinforced by FST estimates, which indicated that among all cultivated accessions, Early cultivars have the least amount of differentiation (FST = 0.12) from the Wild population (Fig. 3a and Supplementary Table 27). Multiple lines of evidence point to the Landrace-AW accessions representing a feral lineage of carrot that has historically

escaped from cultivation and re-established in the wild. Supporting evidence indicates that the Landrace-AW accessions harbor a large fraction of Landrace A alleles, experienced gene flow from wild materials, and exhibit a morphology that resembles that of wild carrots. Also, demosgraphic analysis indicated that Landrace-AW share a population size bottleneck with other cultivated carrot populations which is not expected in wild population (Supplementary Fig. 3). Given the uncertainty regarding the impact of admixture on the genetic makeup of the Landrace-AW accessions on estimates of genetic diversity and selective sweeps, these accessions were excluded from downstream analyses.

7. Selective sweep Enrichment analysis

The enrichment analysis of genes spanning the selective sweep regions were performed using the KEGG [59], TAIR (https://www.arabidopsis.org/), pfam (http://pfam-legacy.xfam.org) and interproscan databases (https://www.ebi.ac.uk/interpro). The GO annotation was performed using WEGO and AgriGO, wherein their significance was assessed with Fisher's exact statistical test [60,61]. The significant gene ontologies and metabolic pathways curation were performed by gene set enrichment analysis using an open-source R Bioconductor package (R v4.3.0; New Jersey, United States) considering Hochberg-FDR adjustment cut-off <0.01 [62].

8. Phenotyping plant material

The phenotypic data for GWA analyses included HPLC data and visual color scores. Visual color scoring was completed for 630 carrot accessions, taking a cross-section of the tap root and assigning categorical scores of white, yellow, orange, red, and purple (Supplementary Table 30). The α-carotene, β-carotene, lutein and lycopene content was measured in 528 accessions within three weeks of harvest. Within two weeks of harvest, slices were taken at mid-root, lyophilized, and processed as in [63,64]. Lyophilized root samples were crushed and 0.1 g of tissue were soaked in 2.0 ml of petroleum ether for 16 hours at 4°C. After soaking, 300 µl of the resulting extract were added to 700 µl of methanol and eluted through a Rainin Microsorb-MV 5 µm column and detected on a Shimadzu SPD-M20A photodiode array detector. Synthetic β-carotene (Sigma [Sigma Chemical], St. Louis, MO) was used as a standard to normalize between independent HPLC runs, and each sample was injected twice during the sample HPLC run to provide technical replication. Lutein, α-carotene, and β-carotene were quantified by absorbance at 450

nm. Carotenoid concentrations was reported in μg/g dry weight of tissue. Since α-carotene and β-carotene are the primary carotenoids in orange carrots, and they account for carrot's orange color [59, 62], the sum of the α-carotene + β-carotene concentration, relative to total carotenoid concentration, on a per sample basis, was used as an objective measure of carrot color in this study. This ratio is denoted as the α-carotene + β-carotene to total carotenoid ratio, or fraction of α-carotene + β-carotene to total carotenoid (Supplementary Table 30). A similar measure of lutein concentration was reported. The HPLC data was filtered to remove samples with inconsistencies between technical replicates. This resulted in a set of 435 accessions with HPLC scores and used for GWA analyses. A Spearman's rank correlation of the ratio of the carotenoids relative to the total carotenoids was completed in base R (v3.6.3) (R Core Team) and plotted with the R package ggplot [65](Supplementary Fig. 8).

9. RNA-sequencing analysis

Experimental Design and RNA Sequencing. In order to profile gene expression in the context of *Or* segregation, seed from an F_3 population derived from a cross between an orange inbred parent (B493) from the USDA carrot breeding program and a white wild carrot (Z007) collected in Uzbekistan (accession Ames 27395) was obtained from [66]. This population was chosen for transcriptome profiling as it was homozygous recessive (based on pedigree and previous mapping information) at known major carotenoid accumulation loci, *Y* and Y_2 [1,67], yet also segregating at the *Or* locus. Seed was planted in a greenhouse at the University of Wisconsin-Madison and harvested at 21-day intervals, starting at 60 days after planting. The first time point was chosen as it corresponded to the visual onset of carotenoid accumulation in the roots, as in [67].

Since this population was segregating for the cultivated *Or* allele (*Or_C*) and the wild *Or* allele (*Or_W)*, root and leaf tissue from each plant were flash-frozen in liquid nitrogen. Primers were designed to PCR-genotype a SNP located in exon 8 of the *Or-like* (DCAR_310369) coding DNA sequence (Supplementary Table 46). Genomic DNA was then extracted from leaf samples and plants were genotyped by Sanger sequencing of the PCR amplicon generated using the primers listed in Supplementary Table 46. Four roots homozygous for *Or_C* and four roots homozygous for *Or_W* were identified at each of the 3 time points, for a total of 24 samples (Supplementary Table 42). Total RNA was extracted from roots using the TRIzol Plus RNA

Purification Kit (Life Technologies, Carlsbad, CA). Genomic DNA contamination was removed using an on-column DNAse digestion (Invitrogen, Carlsbad, CA). RNA integrity was evaluated using an RNA NanoChip on an Agilent 2100 Bioanalyzer. Sequencing libraries were prepared using a TruSeq Stranded mRNA kit (Illumina, San Diego, CA) and libraries were sequenced on a NovaSeq 6000 sequencer at the University of Wisconsin Biotechnology Center in Madison, Wisconsin. Transcriptome sequencing generated 1,091,729,253 reads across 24 samples (Supplementary Table 42).

The interrologous transcriptional interactome network was predicted by using string database and analyzed Cytoscape (v3.9) [68,69]. The genes having conserved orthologs with the plant species network databases having significant correlation edge (FDR ≤ 0.05) were considered as nodes in the predicted network (Supplementary Table 40). The MCODE tool into Cytoscape was used to predict the organ specific enrichment of Gene Ontologies, KEGG pathways and STRING clusters related functional modules in the constructed network [70] (Supplementary Table 41). The expression correlation of predicted network was computed among the nodes using Pearson's correlation to study the co-expression among significantly enriched pathways (FDR ≤ 1e-2) using R Bio-conductor (v4.2.1) package following similar methodologies as carried out in earlier studies [71,72].

10. Genotyping the Y2 insertion

Reads mapping to 3,822 bp long sequence, containing Y2 gene and 1kb long flanking region, were extracted and used for genotyping. To call TE insertion we used custom scripts. In brief, reads were mapped to the analyzed region, in which TE sequence was masked, using bowtie2 (--time --very-fast-local) and part of soft-clipped read that did not match the sequence was extracted, blasted against the Y2 region (-dust no -soft_masking false), and number of reads was reported for the 200 nt long terminal regions of TE. The empty site was supported by identification of part of soft-clipped reads, extracted from result of bowtie2 mapping of all reads to the Y2 region with insertion of TE, in the 400 nt long region flanking TE insertion. If both types of reads were identified, the genotype was called as heterozygous. The minimum. Minimum number of soft-clipped reads was set to 1.

References:

1 Iorizzo, M. *et al.* A high-quality carrot genome assembly provides new insights into carotenoid accumulation and asterid genome evolution. *Nat Genet* **48**, 657-666, doi:10.1038/ng.3565 (2016).

2 Begik, O. *et al.* Quantitative profiling of native RNA modifications and their dynamics using nanopore sequencing. *bioRxiv*, 2020.2007.2006.189969, doi:10.1101/2020.07.06.189969 (2021).

3 Bolger, A. M., Lohse, M. & Usadel, B. Trimmomatic: a flexible trimmer for Illumina sequence data. *Bioinformatics* **30**, 2114-2120, doi:10.1093/bioinformatics/btu170 (2014).

4 Chin, C.-S. *et al.* Phased diploid genome assembly with single-molecule real-time sequencing. *Nature Methods* **13**, 1050-1054, doi:10.1038/nmeth.4035 (2016).

5 Koren, S. *et al.* Canu: scalable and accurate long-read assembly via adaptive k-mer weighting and repeat separation. *Genome Res* **27**, 722-736, doi:10.1101/gr.215087.116 (2017).

6 Walker, B. J. *et al.* Pilon: An Integrated Tool for Comprehensive Microbial Variant Detection and Genome Assembly Improvement. *PLOS ONE* **9**, e112963, doi:10.1371/journal.pone.0112963 (2014).

7 Li, H. & Durbin, R. Fast and accurate short read alignment with Burrows–Wheeler transform. *Bioinformatics* **25**, 1754-1760, doi:10.1093/bioinformatics/btp324 (2009).

8 Ghurye, J. *et al.* Integrating Hi-C links with assembly graphs for chromosome-scale assembly. *PLOS Computational Biology* **15**, e1007273, doi:10.1371/journal.pcbi.1007273 (2019).

9 English, A. C. *et al.* Mind the gap: upgrading genomes with Pacific Biosciences RS long-read sequencing technology. *PLoS One* **7**, e47768, doi:10.1371/journal.pone.0047768 (2012).

10 Dobin, A. *et al.* STAR: ultrafast universal RNA-seq aligner. *Bioinformatics* **29**, 15-21, doi:10.1093/bioinformatics/bts635 (2012).

11 Durand, N. C. *et al.* Juicer Provides a One-Click System for Analyzing Loop-Resolution Hi-C Experiments. *Cell Syst* **3**, 95-98, doi:10.1016/j.cels.2016.07.002 (2016).

12 Robinson, J. T., Thorvaldsdóttir, H., Wenger, A. M., Zehir, A. & Mesirov, J. P. Variant Review with the Integrative Genomics Viewer. *Cancer Research* **77**, e31-e34, doi:10.1158/0008-5472.Can-17-0337 (2017).

13 Robinson, J. T. *et al.* Juicebox.js Provides a Cloud-Based Visualization System for Hi-C Data. *Cell Syst* **6**, 256-258.e251, doi:10.1016/j.cels.2018.01.001 (2018).

14 Alonge, M. *et al.* RaGOO: fast and accurate reference-guided scaffolding of draft genomes. *Genome Biology* **20**, 224, doi:10.1186/s13059-019-1829-6 (2019).

15 Xu, G.-C. *et al.* LR_Gapcloser: a tiling path-based gap closer that uses long reads to complete genome assembly. *GigaScience* **8**, doi:10.1093/gigascience/giy157 (2018).

16 Marçais, G. *et al.* MUMmer4: A fast and versatile genome alignment system. *PLOS Computational Biology* **14**, e1005944, doi:10.1371/journal.pcbi.1005944 (2018).

17 Kent, W. J. *et al.* The human genome browser at UCSC. *Genome Res* **12**, 996-1006, doi:10.1101/gr.229102 (2002).

18 Wingett, S. & Andrews, S. FastQ Screen: A tool for multi-genome mapping and quality control [version 2; peer review: 4 approved]. *F1000Research* **7**, doi:10.12688/f1000research.15931.2 (2018).
19 Bannoud, F. *et al.* Genetic and Transcription Profile Analysis of Tissue-Specific Anthocyanin Pigmentation in Carrot Root Phloem. *Genes (Basel)* **12**, doi:10.3390/genes12101464 (2021).
20 Iorizzo, M. *et al.* Genetic structure and domestication of carrot (Daucus carota subsp. sativus) (Apiaceae). *American Journal of Botany* **100**, 930-938, doi:https://doi.org/10.3732/ajb.1300055 (2013).
21 Pertea, M. *et al.* StringTie enables improved reconstruction of a transcriptome from RNA-seq reads. *Nature Biotechnology* **33**, 290-295, doi:10.1038/nbt.3122 (2015).
22 Wu, T. D. & Watanabe, C. K. GMAP: a genomic mapping and alignment program for mRNA and EST sequences. *Bioinformatics* **21**, 1859-1875, doi:10.1093/bioinformatics/bti310 (2005).
23 Macko-Podgórni, A., Machaj, G. & Grzebelus, D. A Global Landscape of Miniature Inverted-Repeat Transposable Elements in the Carrot Genome. *Genes (Basel)* **12**, 859, doi:10.3390/genes12060859 (2021).
24 Bao, W., Kojima, K. K. & Kohany, O. Repbase Update, a database of repetitive elements in eukaryotic genomes. *Mobile DNA* **6**, 11, doi:10.1186/s13100-015-0041-9 (2015).
25 Neumann, P., Novák, P., Hoštáková, N. & Macas, J. Systematic survey of plant LTR-retrotransposons elucidates phylogenetic relationships of their polyprotein domains and provides a reference for element classification. *Mobile DNA* **10**, 1, doi:10.1186/s13100-018-0144-1 (2019).
26 Novák, P., Neumann, P. & Macas, J. Graph-based clustering and characterization of repetitive sequences in next-generation sequencing data. *BMC Bioinformatics* **11**, 378, doi:10.1186/1471-2105-11-378 (2010).
27 Novák, P., Neumann, P., Pech, J., Steinhaisl, J. & Macas, J. RepeatExplorer: a Galaxy-based web server for genome-wide characterization of eukaryotic repetitive elements from next-generation sequence reads. *Bioinformatics* **29**, 792-793, doi:10.1093/bioinformatics/btt054 (2013).
28 Kwolek, K. *et al.* Diverse and mobile: eccDNA-based identification of carrot low-copy-number LTR retrotransposons active in callus cultures. *The Plant Journal* **110**, 18, doi:https://doi.org/10.1111/tpj.15773 (2022).
29 Ellinghaus, D., Kurtz, S. & Willhoeft, U. LTRharvest, an efficient and flexible software for de novo detection of LTR retrotransposons. *BMC Bioinformatics* **9**, 18, doi:10.1186/1471-2105-9-18 (2008).
30 Miele, V., Penel, S. & Duret, L. Ultra-fast sequence clustering from similarity networks with SiLiX. *BMC Bioinformatics* **12**, 116, doi:10.1186/1471-2105-12-116 (2011).
31 Katoh, K. & Standley, D. M. MAFFT multiple sequence alignment software version 7: improvements in performance and usability. *Mol Biol Evol* **30**, 772-780, doi:10.1093/molbev/mst010 (2013).
32 Paradis, E. & Schliep, K. ape 5.0: an environment for modern phylogenetics and evolutionary analyses in R. *Bioinformatics* **35**, 526-528, doi:10.1093/bioinformatics/bty633 (2018).
33 Ma, J. & Bennetzen, J. L. Rapid recent growth and divergence of rice nuclear genomes. *Proc Natl Acad Sci U S A* **101**, 12404-12410, doi:10.1073/pnas.0403715101 (2004).

34 Wicker, T. & Keller, B. Genome-wide comparative analysis of copia retrotransposons in Triticeae, rice, and Arabidopsis reveals conserved ancient evolutionary lineages and distinct dynamics of individual copia families. *Genome Res* **17**, 1072-1081, doi:10.1101/gr.6214107 (2007).

35 Xiong, W., He, L., Lai, J., Dooner, H. K. & Du, C. HelitronScanner uncovers a large overlooked cache of <i>Helitron</i> transposons in many plant genomes. *Proceedings of the National Academy of Sciences* **111**, 10263-10268, doi:doi:10.1073/pnas.1410068111 (2014).

36 Iorizzo, M. *et al.* De novo assembly and characterization of the carrot transcriptome reveals novel genes, new markers, and genetic diversity. *BMC Genomics* **12**, 389, doi:10.1186/1471-2164-12-389 (2011).

37 Holt, C. & Yandell, M. MAKER2: an annotation pipeline and genome-database management tool for second-generation genome projects. *BMC Bioinformatics* **12**, 491, doi:10.1186/1471-2105-12-491 (2011).

38 Stanke, M. & Morgenstern, B. AUGUSTUS: a web server for gene prediction in eukaryotes that allows user-defined constraints. *Nucleic acids research* **33**, W465-W467, doi:10.1093/nar/gki458 (2005).

39 Pilkington, S. M. *et al.* A manually annotated Actinidia chinensis var. chinensis (kiwifruit) genome highlights the challenges associated with draft genomes and gene prediction in plants. *BMC Genomics* **19**, 257, doi:10.1186/s12864-018-4656-3 (2018).

40 Keilwagen, J., Hartung, F. & Grau, J. GeMoMa: Homology-Based Gene Prediction Utilizing Intron Position Conservation and RNA-seq Data. *Methods Mol Biol* **1962**, 161-177, doi:10.1007/978-1-4939-9173-0_9 (2019).

41 Gremme, G., Brendel, V., Sparks, M. E. & Kurtz, S. Engineering a software tool for gene structure prediction in higher organisms. *Information and Software Technology* **47**, 965-978, doi:https://doi.org/10.1016/j.infsof.2005.09.005 (2005).

42 Conesa, A. *et al.* Blast2GO: a universal tool for annotation, visualization and analysis in functional genomics research. *Bioinformatics* **21**, 3674-3676, doi:10.1093/bioinformatics/bti610 (2005).

43 O'Leary, N. A. *et al.* Reference sequence (RefSeq) database at NCBI: current status, taxonomic expansion, and functional annotation. *Nucleic Acids Res* **44**, D733-745, doi:10.1093/nar/gkv1189 (2016).

44 Ashburner, M. *et al.* Gene ontology: tool for the unification of biology. The Gene Ontology Consortium. *Nat Genet* **25**, 25-29, doi:10.1038/75556 (2000).

45 Kanehisa, M. Toward understanding the origin and evolution of cellular organisms. *Protein Sci* **28**, 1947-1951, doi:10.1002/pro.3715 (2019).

46 Blum, M. *et al.* The InterPro protein families and domains database: 20 years on. *Nucleic Acids Research* **49**, D344-D354, doi:10.1093/nar/gkaa977 (2020).

47 Camon, E. B. *et al.* An evaluation of GO annotation retrieval for BioCreAtIvE and GOA. *BMC Bioinformatics* **6 Suppl 1**, S17, doi:10.1186/1471-2105-6-s1-s17 (2005).

48 Dai, X., Sinharoy, S., Udvardi, M. & Zhao, P. X. PlantTFcat: an online plant transcription factor and transcriptional regulator categorization and analysis tool. *BMC Bioinformatics* **14**, 321, doi:10.1186/1471-2105-14-321 (2013).

49 Osuna-Cruz, C. M. *et al.* PRGdb 3.0: a comprehensive platform for prediction and analysis of plant disease resistance genes. *Nucleic Acids Res* **46**, D1197-d1201, doi:10.1093/nar/gkx1119 (2018).

50 Waterhouse, R. M. *et al.* BUSCO Applications from Quality Assessments to Gene Prediction and Phylogenomics. *Mol Biol Evol* **35**, 543-548, doi:10.1093/molbev/msx319 (2018).
51 Nawrocki, E. P. & Eddy, S. R. Infernal 1.1: 100-fold faster RNA homology searches. *Bioinformatics* **29**, 2933-2935, doi:10.1093/bioinformatics/btt509 (2013).
52 Tardaguila, M. *et al.* SQANTI: extensive characterization of long-read transcript sequences for quality control in full-length transcriptome identification and quantification. *Genome Res* **28**, 396-411, doi:10.1101/gr.222976.117 (2018).
53 Curaba, J. *et al.* Identification of an SCPL Gene Controlling Anthocyanin Acylation in Carrot (Daucus carota L.) Root. *Frontiers in Plant Science* **10**, doi:10.3389/fpls.2019.01770 (2020).
54 Livak, K. J. & Schmittgen, T. D. Analysis of relative gene expression data using real-time quantitative PCR and the 2(-Delta Delta C(T)) Method. *Methods* **25**, 402-408, doi:10.1006/meth.2001.1262 (2001).
55 Tian, J. *et al.* Real-Time Automatic Segmentation of Optical Coherence Tomography Volume Data of the Macular Region. *PLOS ONE* **10**, e0133908, doi:10.1371/journal.pone.0133908 (2015).
56 Ranson, H., Collins, F. & Hemingway, J. The role of alternative mRNA splicing in generating heterogeneity within the Anopheles gambiae class I glutathione S-transferase family. *Proc Natl Acad Sci U S A* **95**, 14284-14289, doi:10.1073/pnas.95.24.14284 (1998).
57 Jiang, S. Y., Jin, J., Sarojam, R. & Ramachandran, S. A Comprehensive Survey on the Terpene Synthase Gene Family Provides New Insight into Its Evolutionary Patterns. *Genome Biol Evol* **11**, 2078-2098, doi:10.1093/gbe/evz142 (2019).
58 Fitak, R. R. OptM: estimating the optimal number of migration edges on population trees using Treemix. *Biology Methods and Protocols* **6**, bpab017, doi:10.1093/biomethods/bpab017 (2021).
59 Kanehisa, M., Sato, Y., Furumichi, M., Morishima, K. & Tanabe, M. New approach for understanding genome variations in KEGG. *Nucleic Acids Research* **47**, D590-D595, doi:10.1093/nar/gky962 (2018).
60 Ye, J. *et al.* WEGO 2.0: a web tool for analyzing and plotting GO annotations, 2018 update. *Nucleic Acids Research* **46**, W71-W75, doi:10.1093/nar/gky400 (2018).
61 Du, Z., Zhou, X., Ling, Y., Zhang, Z. & Su, Z. agriGO: a GO analysis toolkit for the agricultural community. *Nucleic Acids Research* **38**, W64-W70, doi:10.1093/nar/gkq310 (2010).
62 Benjamini, Y. & Hochberg, Y. Controlling the False Discovery Rate: A Practical and Powerful Approach to Multiple Testing. *Journal of the Royal Statistical Society: Series B (Methodological)* **57**, 289-300, doi:https://doi.org/10.1111/j.2517-6161.1995.tb02031.x (1995).
63 Simon, P. *et al.* High carotene mass carrot population. (1989).
64 Simon, P. W. & Wolff, X. Y. Carotenes in typical and dark orange carrots. *Journal of Agricultural and Food Chemistry* **35**, 1017-1022, doi:10.1021/jf00078a038 (1987).
65 Wickham, H. *ggplot2: Elegant Graphics for Data Analysis*. (Springer International Publishing, 2016).

66 Coe, K. M., Ellison, S., Senalik, D., Dawson, J. & Simon, P. The influence of the Or and Carotene Hydroxylase genes on carotenoid accumulation in orange carrots [Daucus carota (L.)]. *Theor Appl Genet* **134**, 3351-3362, doi:10.1007/s00122-021-03901-3 (2021).
67 Ellison, S., Senalik, D., Bostan, H., Iorizzo, M. & Simon, P. Fine Mapping, Transcriptome Analysis, and Marker Development for Y(2) , the Gene That Conditions β-Carotene Accumulation in Carrot (Daucus carota L.). *G3 (Bethesda)* **7**, 2665-2675, doi:10.1534/g3.117.043067 (2017).
68 Shannon, P. *et al.* Cytoscape: a software environment for integrated models of biomolecular interaction networks. *Genome Res* **13**, 2498-2504, doi:10.1101/gr.1239303 (2003).
69 Szklarczyk, D. *et al.* STRING v10: protein-protein interaction networks, integrated over the tree of life. *Nucleic Acids Res* **43**, D447-452, doi:10.1093/nar/gku1003 (2015).
70 Bader, G. D. & Hogue, C. W. V. An automated method for finding molecular complexes in large protein interaction networks. *BMC Bioinformatics* **4**, 2, doi:10.1186/1471-2105-4-2 (2003).
71 Seth, R. *et al.* An Integrative Transcriptional Network Revealed Spatial Molecular Interplay Underlying Alantolactone and Inulin Biosynthesis in Inula racemosa Hook f. *Int J Mol Sci* **23**, doi:10.3390/ijms231911213 (2022).
72 Seth, R., Maritim, T. K., Parmar, R. & Sharma, R. K. Underpinning the molecular programming attributing heat stress associated thermotolerance in tea (Camellia sinensis (L.) O. Kuntze). *Horticulture Research* **8**, 99, doi:10.1038/s41438-021-00532-z (2021).

Supplementary Figures

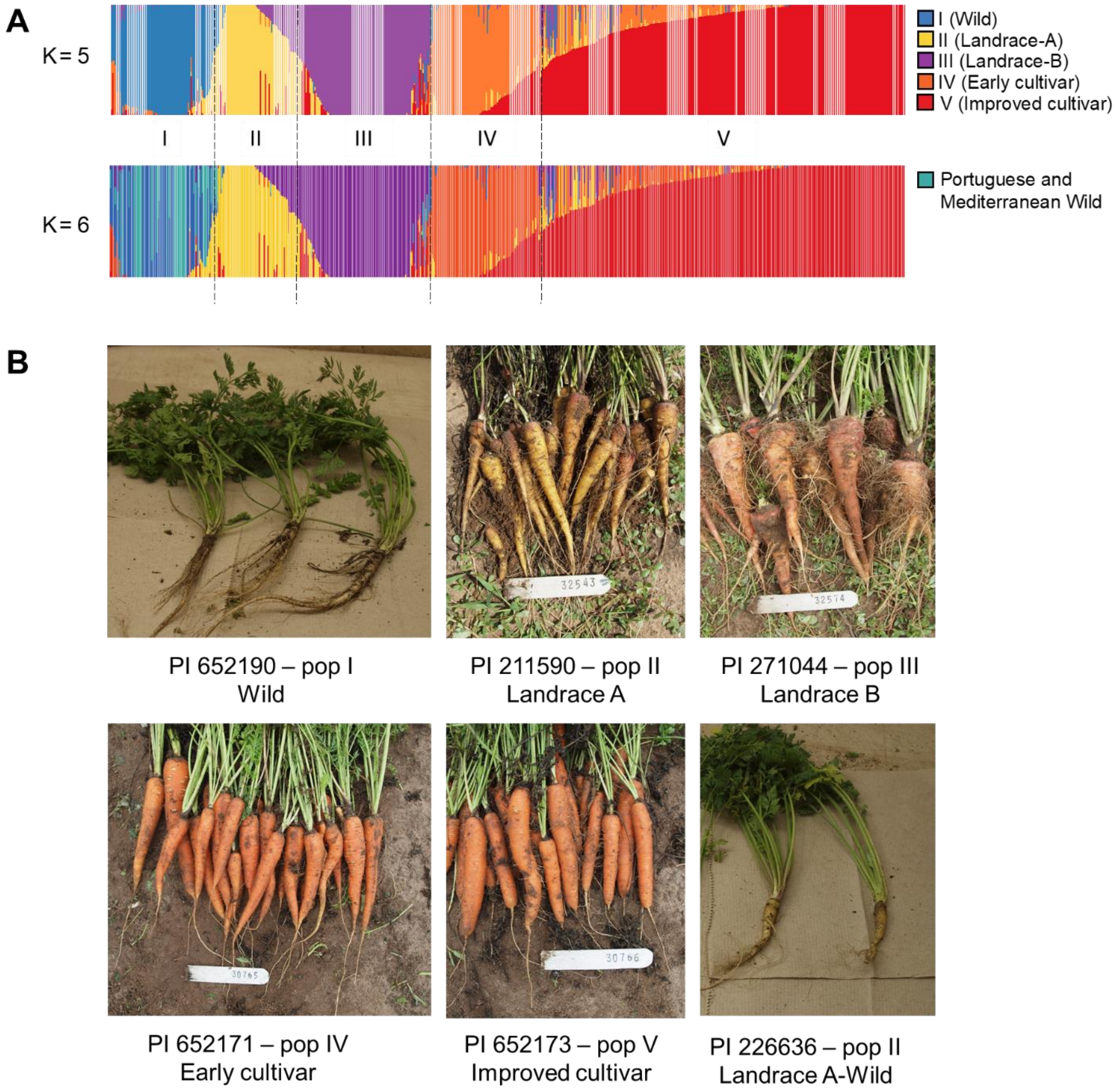

Supplementary Figure 1. Model-based Population Structure of 630 resequenced carrot accessions. A) Barplot of admixture proportions at K = 5 and K=6. B) Accession’s phenotypes representative of each population at K5.

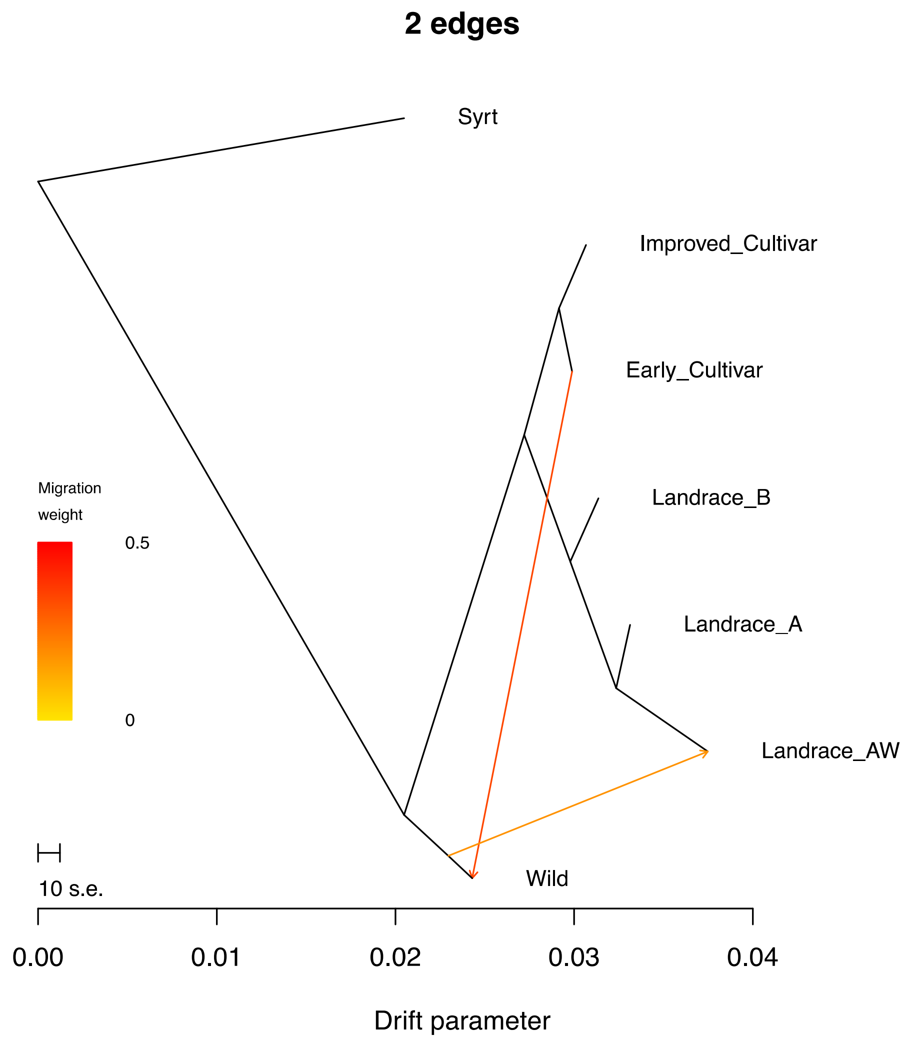

Supplementary Figure 2. TreeMix output for 2 migration edges. Migration edges are shaded based on the migration weight.

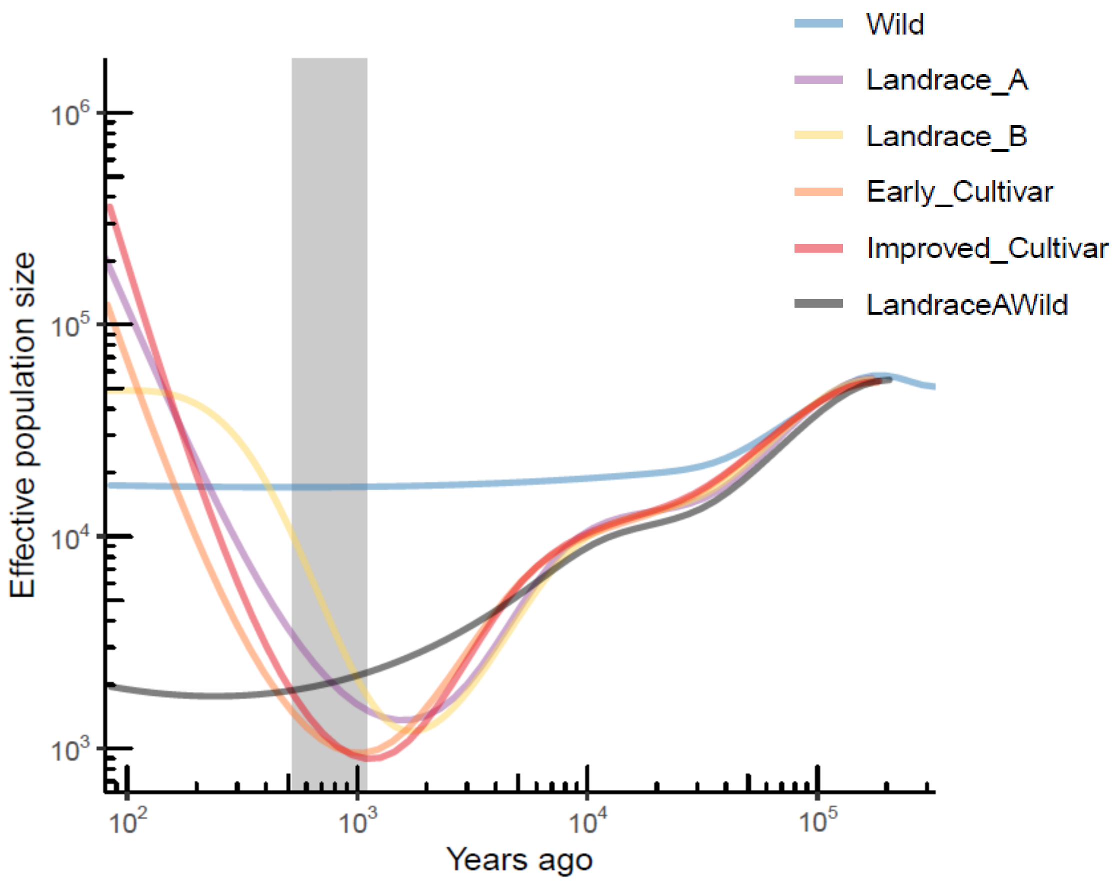

Supplementary Figure 3. Demographic history inferred by including the landrace A-wild accessions.

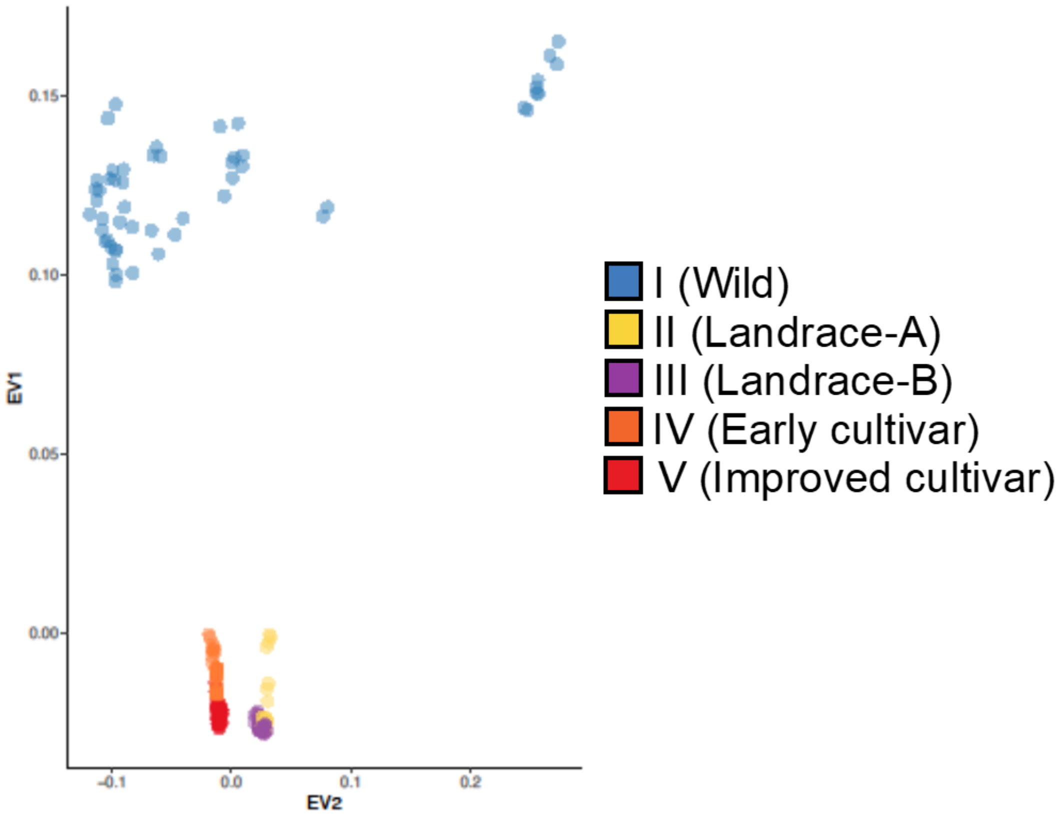

Supplementary Figure 4. PCA of Low Admixture Samples.

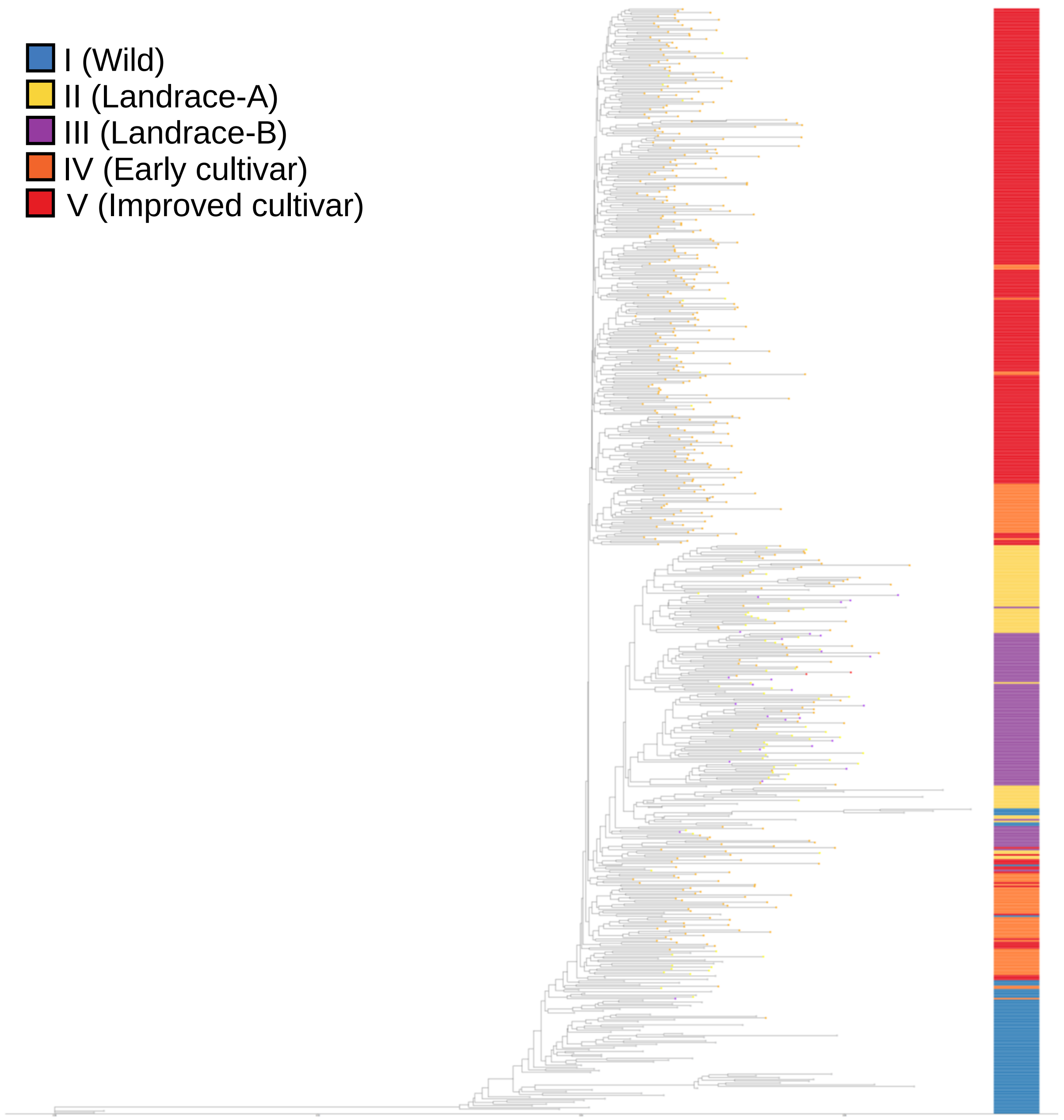

Supplementary Figure 5. Neighbor joining phylogenetic tree of 630 samples. The consensus tree was constructed with 100 boostrap replicates. Branch tip colors represent the root color phenotypes and the outer bar corresponds to the population identity of each sample. The tree was rooted using *D. syrticus* as the outgroup.

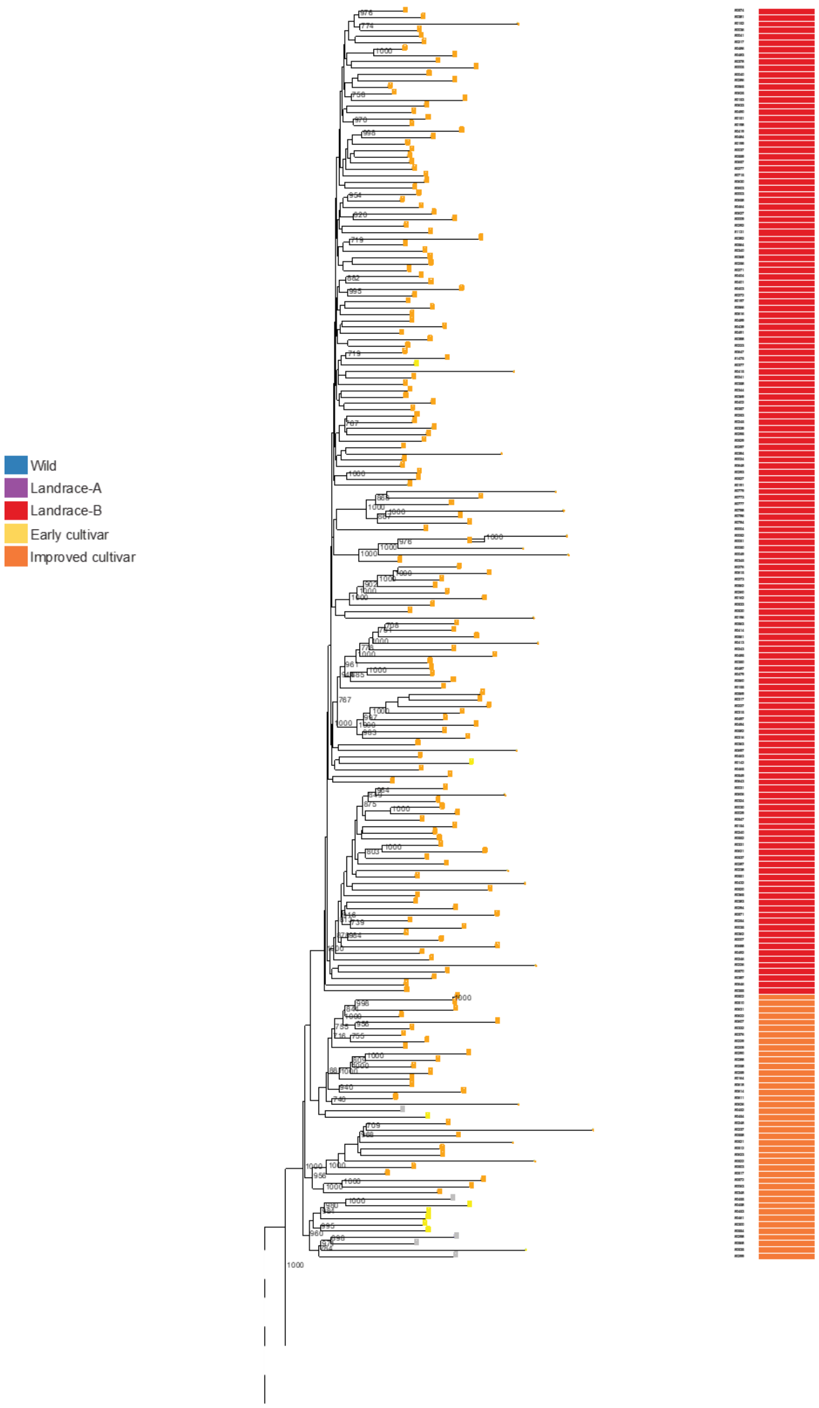

Supplementary Figure 6. Neighbor joining phylogenetic tree of the low admixture set. The consensus tree was constructed with 1,000 boostrap replicates. Branch tip colors represent the root color phenotypes and the outer bar corresponds to the population identity of each sample. The tree was rooted using *D. syrticus* as the outgroup. Bootstrap value >70% support are illustrated.

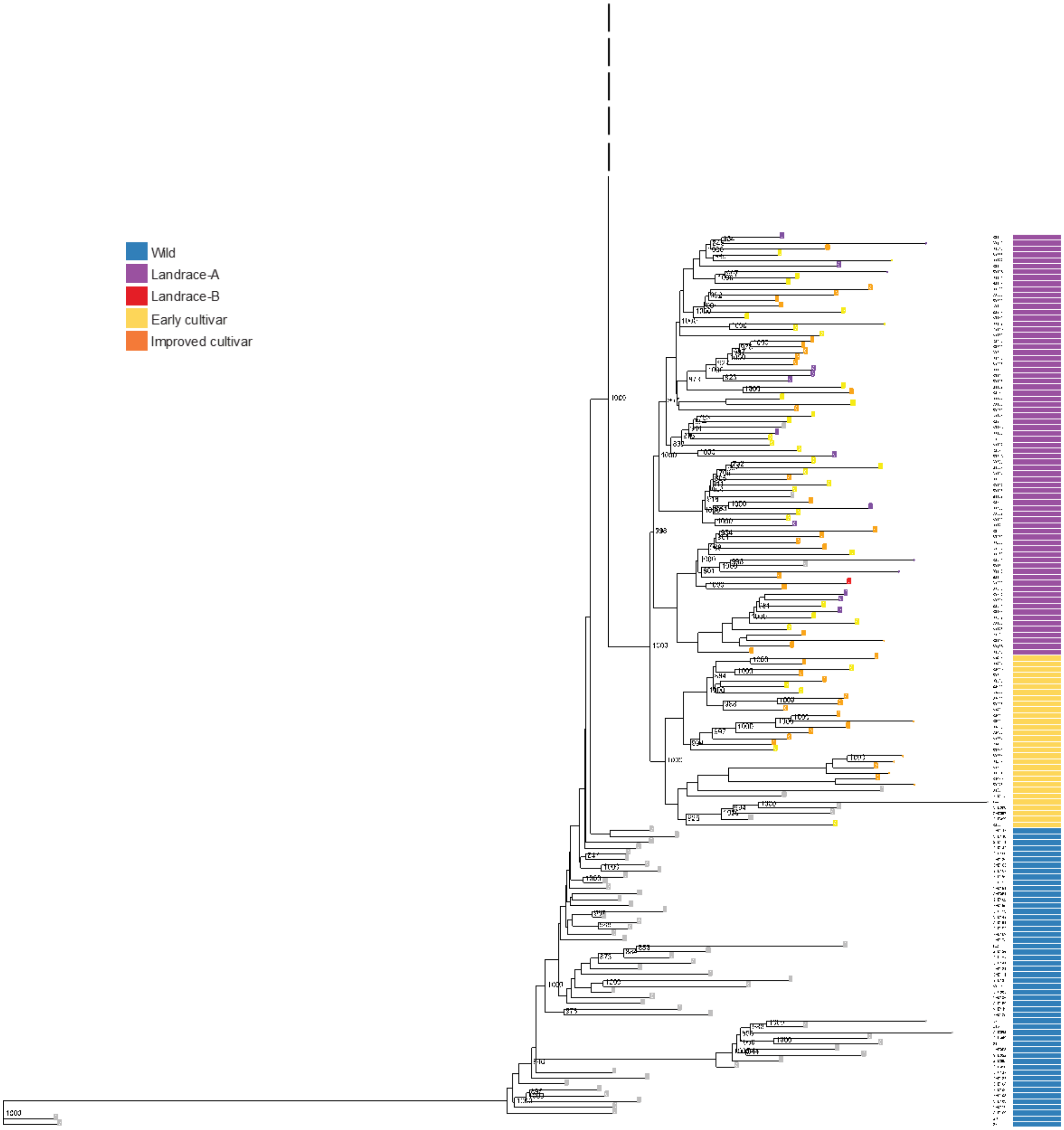

Continue Supplementary Figure 6. Neighbor joining phylogenetic tree of the low admixture set. The consensus tree was constructed with 1,000 boostrap replicates. Branch tip colors represent the root color phenotypes and the outer bar corresponds to the population identity of each sample. The tree was rooted using *D. syrticus* as the outgroup. Bootstrap value >70% support are illustrated.

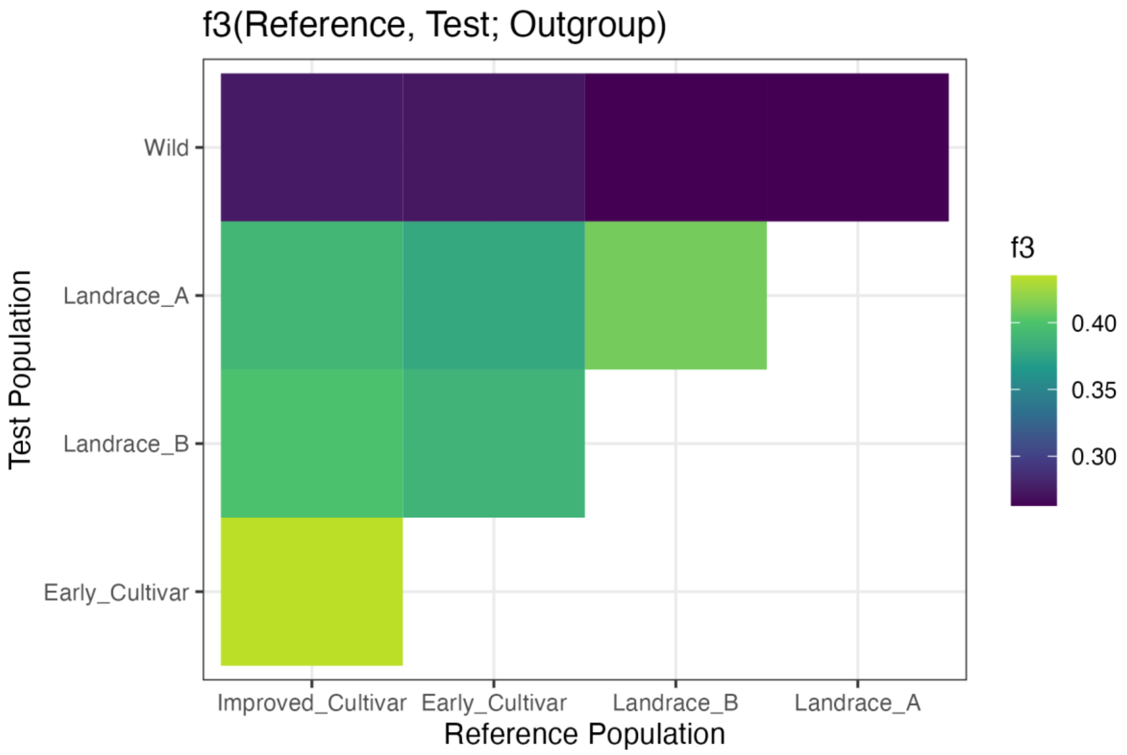

Supplementary Figure 7. Values for population comparisons using f3-outgroup statistics as a measure of shared genetic drift between pairs of populations relative to a distantly related outgroup. Tests were represented as f3(reference population, test population; outgroup). The outgroup population contained wild samples from *Daucus carota* subspecies (subsp. *gummifer*, *maximus carota*, *and maritimus carota*), which are genetically equidistant to the test and reference populations. Higher f3 values indicate a higher degree of genetic similarity and a longer shared branch length between the reference and test populations relative to the outgroup.

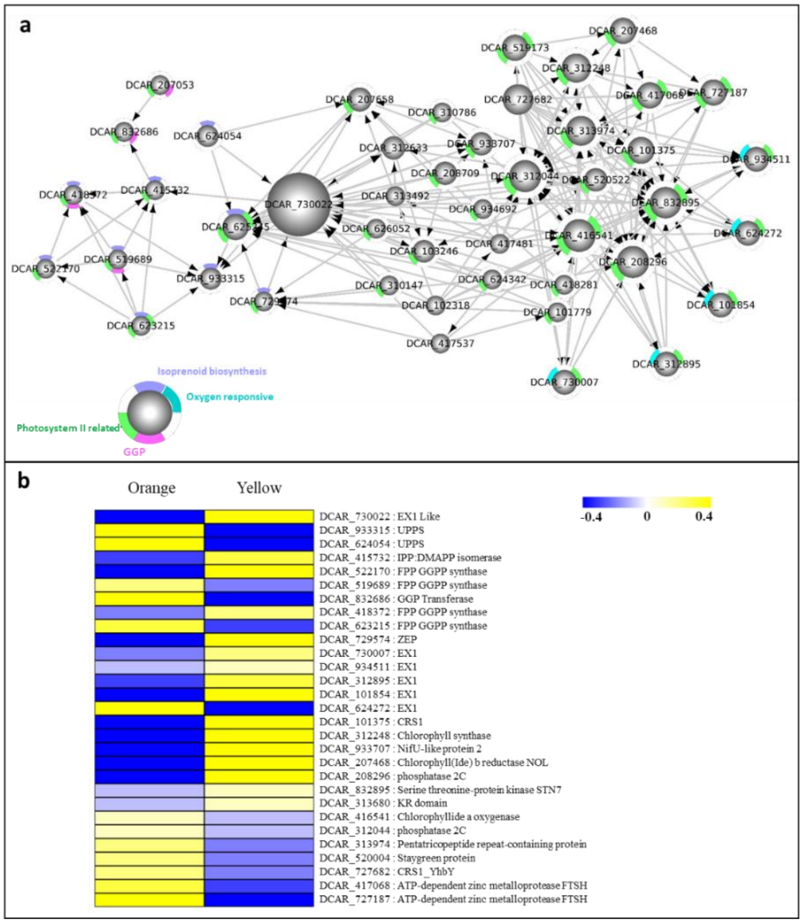

Supplementary Figure 8. Interactome and enrichment analysis for gene DCAR_730022. a) Predicted Transcriptional interactome network representing significant interaction of DCAR_730022 with gene corresponding to photosystem II including response to singlet oxygen (DCAR_730007, DCAR_934511, DCAR_312895, DCAR_101854, DCAR_624272) and isoprenoid biosynthesis pathways (DCAR_933315, DCAR_624054, DCAR_415732, DCAR_522170, DCAR_519689, DCAR_832686, DCAR_418372, DCAR_623215) analyzed and visualized using Cytoscape. b) Heatmap representing expression-based enrichment (based on z-score value) of key genes having significant interactions with DCAR_730022 in the predicted. The significant interactions were observed between the DCAR_730022 with the genes involved in both Isoprenoid biosynthesis and photosystem related genes. In particular the gene has a significant interaction with DCAR_933315 (Undecaprenyl pyrophosphate synthase (UPPs) involved in the synthesis of Farnesyl Pyrophosphate (FPP) synthesis) and genes involved in GGP synthesis (DCAR_519689 & DCAR_832686) which act as precursor during carotenoid biosynthesis.

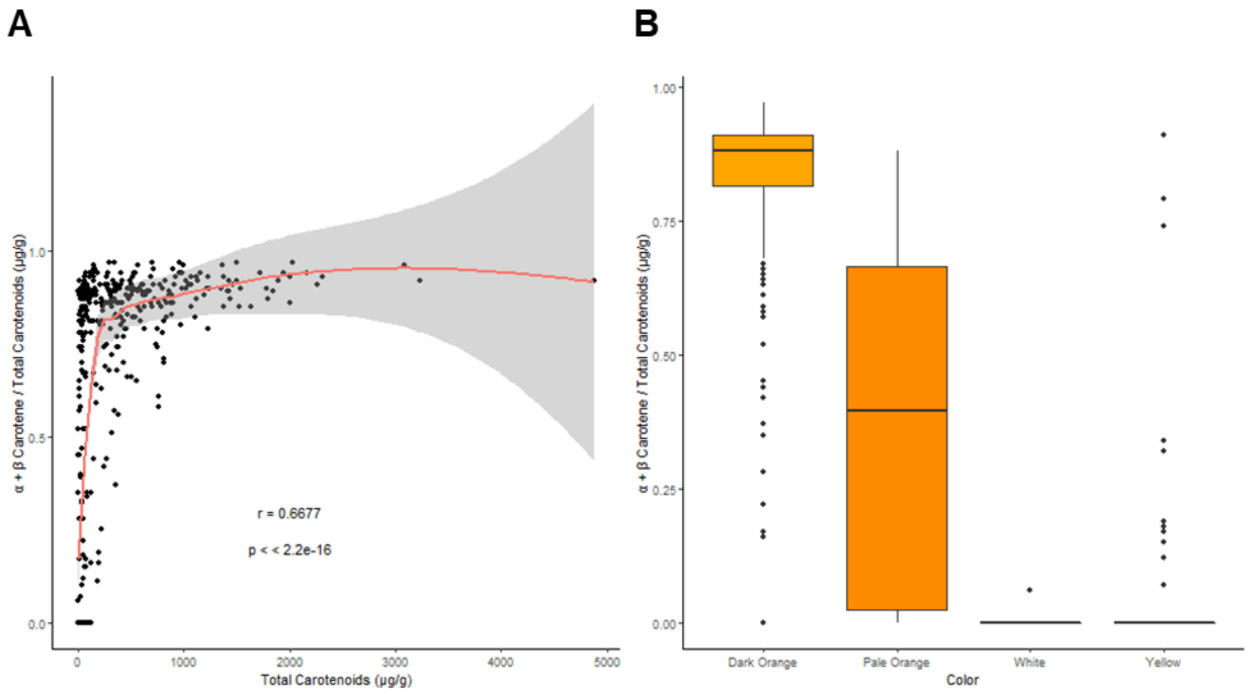

Supplementary Figure 9. Relationship between total carotenoid content and % of α- + β-carotene. A) Correlation between the ratio of α- + β-carotene concentration to total carotenoid concentration, or ABCR (ordinate) and the total carotenoid concentration (abscissa) in the carrot accessions evaluated in this study. Red and purple samples (n=29) were not included in this analysis. Based on Spearman's correlation, carotenoid concentration is strongly correlated with the ratio of α and β-carotene (two-sided, p = 1.277345e-53). B) The ABCR found in carrots visually phenotyped as dark orange (*n*=279), pale orange (*n*=38), white (*n*=20), and yellow (n=69), categories. Dark orange carrots had a significantly higher ratio of α and β-carotene (LSD, p-value < 0.001) than the other categories. Pale orange carrots also had a significantly higher (LSD, < p-value < 0.001) ratio of α- + β-carotene, relative to yellow and white carrots. Most yellow and all white carrots had a small ratio α and β-carotene and were not statistically different from each other. The boxplot represents the 25th, 50th and 75th percentiles with the upper and lower whisker 1.5x the 75th and 25th percentiles, respectively.

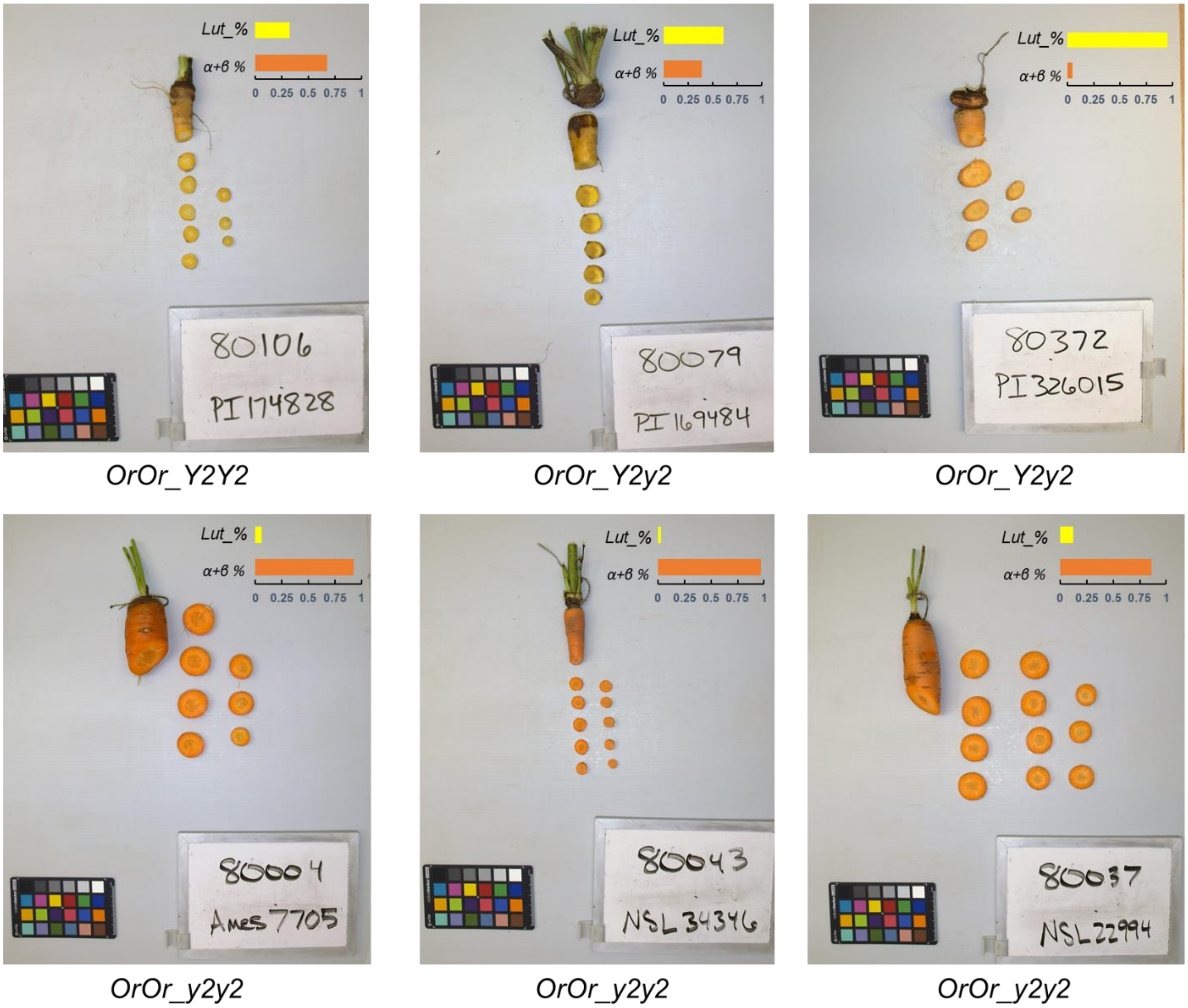

Supplementary Figure 10. Examples of pale orange (pOr) and dark Orange (dOr) phenotype harboring different allele combinations at the *Or* and *EX1* gene. pOr sample harbor a combination of wild and cultivated alleles at the two genes (e.g. A_B_) and dOr genotype harbored cultivated (A_A_) alleles at both genes. The graph illustrated in the up left corner of each picture represent the percentage of lutein and α- +β-carotene relative to the total amount of carotenoids.

References

1 Bannoud, F. *et al.* Genetic and Transcription Profile Analysis of Tissue-Specific Anthocyanin Pigmentation in Carrot Root Phloem. *Genes (Basel)* **12**, doi:10.3390/genes12101464 (2021).
