# Supplementary material for: Population genomics identifies genetic signatures of carrot domestication and improvement and uncovers the origin of high-carotenoid orange carrots
Source: Nat Plants. 2023 Sep 28;9(10):1643–58. doi: 10.1038/s41477-023-01526-6 (PMC10581907; doi:10.1038/s41477-023-01526-6)
Supplement: Supplementary file 2 — Reporting Summary [file 41477_2023_1526_MOESM2_ESM.pdf]

Corresponding author(s): Massimo Iorizzo and Philip Simon

Last updated by author(s): Mar 24, 2023

Reporting Summary

Nature Portfolio wishes to improve the reproducibility of the work that we publish. This form provides structure for consistency and transparency in reporting. For further information on Nature Portfolio policies, see our Editorial Policies and the Editorial Policy Checklist.

Statistics

For all statistical analyses, confirm that the following items are present in the figure legend, table legend, main text, or Methods section.

n/a	Confirmed	
☐	☒	The exact sample size (*n*) for each experimental group/condition, given as a discrete number and unit of measurement
☐	☒	A statement on whether measurements were taken from distinct samples or whether the same sample was measured repeatedly
☐	☒	The statistical test(s) used AND whether they are one- or two-sided<br>*Only common tests should be described solely by name; describe more complex techniques in the Methods section.*
☒	☐	A description of all covariates tested
☒	☐	A description of any assumptions or corrections, such as tests of normality and adjustment for multiple comparisons
☐	☒	A full description of the statistical parameters including central tendency (e.g. means) or other basic estimates (e.g. regression coefficient) AND variation (e.g. standard deviation) or associated estimates of uncertainty (e.g. confidence intervals)
☐	☒	For null hypothesis testing, the test statistic (e.g. *F*, *t*, *r*) with confidence intervals, effect sizes, degrees of freedom and *P* value noted<br>*Give P values as exact values whenever suitable.*
☒	☐	For Bayesian analysis, information on the choice of priors and Markov chain Monte Carlo settings
☐	☒	For hierarchical and complex designs, identification of the appropriate level for tests and full reporting of outcomes
☒	☐	Estimates of effect sizes (e.g. Cohen's *d*, Pearson's *r*), indicating how they were calculated

Our web collection on statistics for biologists contains articles on many of the points above.

Software and code

Policy information about availability of computer code

Data collection	No commercial, open source and custom code were used to collect the data
Data analysis	Specific open source programs used for data analysis are all cited in the manuscript. Also a GitHub page was developed to list software and parameters used in the manuscript. https://github.com/dsenalik/Carrot_Genome_DH1_v3

For manuscripts utilizing custom algorithms or software that are central to the research but not yet described in published literature, software must be made available to editors and reviewers. We strongly encourage code deposition in a community repository (e.g. GitHub). See the Nature Portfolio guidelines for submitting code & software for further information.

Data

Policy information about availability of data

All manuscripts must include a data availability statement. This statement should provide the following information, where applicable:

- Accession codes, unique identifiers, or web links for publicly available datasets
- A description of any restrictions on data availability
- For clinical datasets or third party data, please ensure that the statement adheres to our policy

Data and materials availability: The DH1 v3 genome is available at CarrotOmics.org 124. All sequence data generated for this study were deposited in NCBI, under the umbrella BioProject PRJNA285926. Component BioProjects consist of PRJNA798760 for reads used in the genome assembly, PRJNA865166 for RNAseq BioSamples and reads, and PRJNA865653 for resequenced BioSamples and reads. Assembled genome sequences are available as accession numbers CP093343

through CP093353. Previously published reads used in this study are also available from the umbrella BioProject. Specific BioProject, BioSample, and SRA accessions are also listed in the Supplementary Tables where additional details for each dataset are provided. Lunar White nucleotide sequence were deposited in NCBI under the name, BankIt2620219 lunar_white_DCAR_730022_region accession #OP407851. Also, the list of the software and parameters used in this study were made available through GitHub https://github.com/dsenalik/Carrot_Genome_DH1_v3.

Human research participants

Policy information about studies involving human research participants and Sex and Gender in Research.

Reporting on sex and gender	N/A
Population characteristics	N/A
Recruitment	N/A
Ethics oversight	N/A

Note that full information on the approval of the study protocol must also be provided in the manuscript.

Field-specific reporting

Please select the one below that is the best fit for your research. If you are not sure, read the appropriate sections before making your selection.

☒ Life sciences ☐ Behavioural & social sciences ☐ Ecological, evolutionary & environmental sciences

For a reference copy of the document with all sections, see nature.com/documents/nr-reporting-summary-flat.pdf

Life sciences study design

All studies must disclose on these points even when the disclosure is negative.

Sample size	No specific statistical analysis was conducted to establish samples size. For the re-sequeincing, the number of lines was chosen to maximize the representation of the carrot populations and global geographic regions, which enabled us to maximize the diversity represented in the study. The number of accession used in the study, N=630, is above the typical number of accessions used in similar studies.
Data exclusions	no data was excluded
Replication	The quality of the genome assembly was tested using multiple methods that are described in the supplementary note. This include use of Hi-C data, BAC-end sequences, independently developed linkage maps and transcriptome data. identification of selective sweeps was tested using three methods and only selective sweeps detected with all three methods were considered for analysis in the paper. GWAS analysis for related traits (carotenoid content and color) was performed with HPLC and visual scores, and identification of overlapping QTLs proved the confidence of the QTLs. Robustness of phylogenetic analysis was tested using bootstrap test. Multiple analysis were used to test for gene flow (TreeMix, f4-statistics) and population divergence and effective population size history (f3-statistics, SMC++) and served to validate results.
Randomization	N/A
Blinding	Blinding was not necessary for this study because the main goal was to maximize represetantion of the global carrot germplasm and to avoid sample duplications.

Reporting for specific materials, systems and methods

We require information from authors about some types of materials, experimental systems and methods used in many studies. Here, indicate whether each material, system or method listed is relevant to your study. If you are not sure if a list item applies to your research, read the appropriate section before selecting a response.

Materials & experimental systems

n/a	Involved in the study	
☒	☐	Antibodies
☒	☐	Eukaryotic cell lines
☒	☐	Palaeontology and archaeology
☒	☐	Animals and other organisms
☒	☐	Clinical data
☒	☐	Dual use research of concern

Methods

n/a	Involved in the study	
☒	☐	ChIP-seq
☒	☐	Flow cytometry
☒	☐	MRI-based neuroimaging
